# Chromosome-level genome assemblies from two sandalwood species provide insights into the evolution of the Santalales

**DOI:** 10.1038/s42003-023-04980-2

**Published:** 2023-06-01

**Authors:** Zhou Hong, Dan Peng, Luke R. Tembrock, Xuezhu Liao, Daping Xu, Xiaojin Liu, Zhiqiang Wu

**Affiliations:** 1 Research Institute of Tropical Forestry, Chinese Academy of Forestry, 510520 Guangzhou, China; 2 College of Agriculture, Center for Genomics and Biotechnology, Fujian Agriculture and Forestry University, 350002 Fuzhou, China; 3 Shenzhen Branch, Guangdong Laboratory for Lingnan Modern Agriculture, Genome Analysis Laboratory of the Ministry of Agriculture, Agricultural Genomics Institute at Shenzhen, 518120 Shenzhen, China; 4 Kunpeng Institute of Modern Agriculture at Foshan, Shenzhen Branch, Guangdong Laboratory of Lingnan Modern Agriculture, Agricultural Genomics Institute at Shenzhen, Chinese Academy of Agricultural Sciences, 518124 Shenzhen, China; 5 Department of Agricultural Biology, Colorado State University, Fort Collins, Colorado 80523 USA

**Keywords:** Plant evolution, Phylogenetics

## Abstract

Sandalwood is one of the most expensive woods in the world and is well known for its long-lasting and distinctive aroma. In our study, chromosome-level genome assemblies for two sandalwood species (*Santalum album* and *Santalum* *yasi*) were constructed by integrating NGS short reads, RNA-seq, and Hi-C libraries with PacBio HiFi long reads. The *S. album* and *S. yasi* genomes were both assembled into 10 pseudochromosomes with a length of 229.59 Mb and 232.64 Mb, containing 21,673 and 22,816 predicted genes and a repeat content of 28.93% and 29.54% of the total genomes, respectively. Further analyses resolved a *Santalum*-specific whole-genome triplication event after divergence from ancestors of the Santalales lineage *Malania*, yet due to dramatic differences in transposon content, the *Santalum* genomes were only one-sixth the size of the *Malania oleifera* genome. Examination of RNA-seq data revealed a suite of genes that are differentially expressed in haustoria and might be involved in host hemiparasite interactions. The two genomes presented here not only provide an important comparative dataset for studying genome evolution in early diverging eudicots and hemiparasitic plants but will also hasten the application of conservation genomics for a lineage of trees recovering from decades of overexploitation.

## Abstract (web summary)

Genome assemblies for two species of sandalwood tree, *Santalum album* and *Santalum yasi*, are presented.

## Introduction

*Santalum* (commonly referred to as sandalwood) is a genus of medium-sized evergreen trees and shrubs in the Santalaceae family. Several species of *Santalum* are known for producing highly aromatic wood that has historically been used to produce incense, medicines, and fine woodwork. The geographic range of the genus includes much of southeast Asia, Australia, and many islands in the Pacific where endemic species have evolved^1^. The use and trade of sandalwood has a long history and has been much revered by numerous cultures in Asia, especially India and China^2,3^. Sandalwood is among the most highly valued wood in the world due to its unique characteristics, the rarity of species in the wild, and the difficulty in cultivating trees for mass production of wood^4^. Some of the difficulty in cultivating sandalwood comes from the fact that sandalwoods are hemiparasites that require host trees for the acquisition of mineral resources via haustorial connections^5,6^. To better study, utilize, and preserve the valuable resources of this genus, whole-genome sequencing is an essential first step.

The species *Santalum album* and *S. yasi* are the most important sources of sandalwood, of which *S. album* is the only species found on the Asian mainland, while *S. yasi* is native to the islands of Fiji and Tonga^7^. However, where these species are grown together in plantations, hybridization is known to occur with subsequent changes in metabolic profiles among hybrids^8^. The quality of wood in different sandalwood species has been associated with differences in metabolites. For instance, high levels of α-santalol and β-santalol are present in *S. album* and *S. yasi*, while these compounds are less abundant in other sandalwood species. The production of these aromatic compounds starts with the conversion of Farnesyl diphosphate into a blend of α-santalol, β-santalol, epi-β-santalol, and α-exo-bergamotol by a multi-product sesquiterpene synthase, *santalene synthase* (*SaSSY*), and a plant CYP736A sub-family member, *SaCYP736A167*^9^. Furthermore, *S. yasi* contains careen and santolinatrine, which have not been detected in *S. album*^10^. Several studies have been carried out on the functional genetics of aroma substances in *S. album*. In one study, *SaDXR* was found to control the flux of sandalwood sesquiterpenes and to be related to photosynthetic pigment synthesis^11^. In other studies, the genes *SaTPS1* to *3* and *SaWRKY1* were crucial in the production and accumulation of essential oil, and all of them were regulated by *SA* and *MeJA* in response to biotic and abiotic stresses^12,13^. Understanding the pathways by which these compounds are produced could lead to the development of improved sandalwood cultivars through marker-assisted breeding and/or genetic transformation. In addition to the development of improved sandalwood cultivars, the production of high-quality genomic resources is essential in the implementation of conservation strategies to preserve populations impacted by overharvesting^14^.

Previously, two short-read-based genomes of *S. album* have been published, but the assemblies were fragmented due to limitations of the sequencing and assembly technology at the time^15,16^. As such, there remains a need for more complete genomes to improve phylogenomic studies among sandalwoods and related species. Here, we present a chromosome-level genome assembly for *S. album* and a sandalwood species with no previous complete genome, *S. yasi*, by combining short read and long read HiFi sequencing with Hi-C scaffolding, which are the first such chromosome-scale genomes in the family Santalaceae. Given the early diverging position of Santalales in Superasterids, these genomes will also be an important contribution to the study of plant genome evolution in core eudicots as well as the abovementioned metabolic and conservation-related studies. To this point, we identified a recent Santalaceae-specific whole-genome triplication (WGT) event that occurred after the divergence of Santalaceae from the ancestors of *M. oleifera* using comparative and evolutionary analyses, which provide a basis for future studies on the evolution of Santalales genomes.

## Results

### Genome sequencing and assembly

**Fig. 1:**
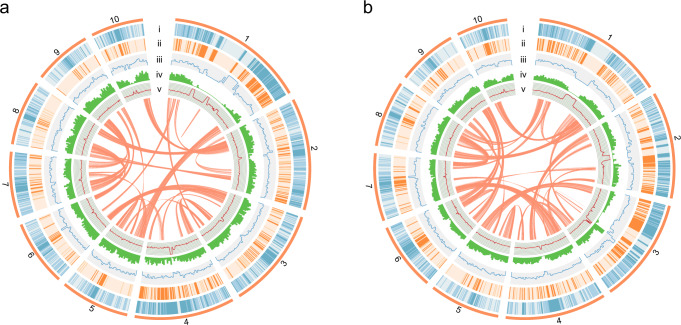
Genomes of *S. album* and *S. yasi* assembled. Distribution of **a** *S. album* and **b** *S. yasi* genomic features. Circular representation of the 10 pseudochromosomes. Tracks i–v represent the *copia* element density, *gypsy* element density, distribution of rRNA, gene density, and GC content, respectively. These metrics were calculated in 200 Kb windows. The paths at the center of the figures represent collinear blocks between chromosomes containing 15 or more genes.

We sequenced and assembled genomes for the sandalwood species *S. album* and *S. yasi* (Fig. 1). In total, ~23 Gb and ~25 Gb of clean short reads of *S. album* and *S. yasi* were obtained for the genomic survey, respectively (Supplementary Tables 1 and 2). According to k-mer analysis, the genome size of *S. album* was estimated to be 225.30 Mb, the heterozygosity was 0.36%, and the repeat content was 24.0% (Supplementary Fig. 1a). The genome size of *S. yasi* was estimated to be 233.18 Mb, the heterozygosity was 0.31%, and the repeat content was 27.6% (Supplementary Fig. 1b). A total of 15 Gb and 10 Gb of HiFi reads were generated for *S. album* and *S. yasi*, with an average read length of 17.98 Kb and 21.59 Kb, and an N50 of 17.84 Kb and 22.17 Kb, respectively (Supplementary Table 1). These HiFi reads were used for initial genome assembly. A draft genome of 258.27 Mb in size with 632 contigs for *S. album* and 273.90 Mb in size with 698 contigs for *S. yasi* were obtained, and a contig N50 of 9.92 Mb and 2.78 Mb, respectively. We used 130x depth valid Hi-C reads to scaffold the contigs into 10 super scaffolds with juicer to obtain 90.78% coverage of the genome of *S. album* (Supplementary Table 3). The final genome contained 10 super scaffolds and 44 contigs with a total length of 229.59 Mb. The size of the contig and scaffold N50 was 10.64 Mb and 26.51 Mb, respectively (Table 1). For *S. yasi*, we used 104x depth valid Hi-C reads of *S. yasi* to assemble the genome into 10 super scaffolds and 56 contigs with a size of 232.64 Mb, and a contig and scaffold N50 of 3.63 Mb and 18.16 Mb, respectively (Table 1). The GC ratios in the two genomes ranged from 36 to 39% throughout much of the genome, with higher GC content in the putative centromeric regions (Fig. 1a, b). To verify the completeness of the *S. album* and *S. yasi* genomes, Benchmarking Universal Single-Copy Orthologs (BUSCO) analyses were conducted and indicated 96.5% and 96.7% completeness, respectively (Supplementary Fig. 2). In addition, the LTR assembly index (LAI) of *S. album* and *S. yasi* was 30.81 and 16.68, respectively, further indicating the high quality of the two sandalwood genomes (Supplementary Fig. 3).

**Table 1:** The overall statistics of the *S. album* and *S. yasi* genome.

Assembly feature		*S. album*	*S. yasi*
Assembly size (Mb)		229.59	232.62
Contig assembly	No. contigs	182	432
	Contig N50 length (Mb)	5.93	1.52
Superscaffold assembly	Anchored size (Mb)	219.49	213.19
	No. scaffolds	54	66
	Scaffold N50 length (Mb)	26.51	18.16
	Contig N50 length (Mb)	10.64	3.63
	Largest scaffold (Mb)	36.92	34.13
	BUSCO (%)	96.5	96.7
	Gene in Superscaffold	21,673	22,816
	Function gene number	20,856	21,045
	Repeat content (%)	28.93	29.54
	BUSCO (%)	93.6	94.3

### Genome annotation

A total of 28.93% of the *S. album* genome was found to be repetitive sequences using the EDTA pipeline (Table 2). Unlike many other plant genomes sequenced thus far, the proportion of LTR retrotransposons in the *S. album* genome was not the repetitive type with the highest proportion and accounted for only 8.09% of the genome, of which *copia* accounted for 6.10% and *gypsy* for 1.09% of the total genome. Both *copia* and *gypsy* LTRs in *S. album* and *S. yasi* were found in higher abundance in the central regions of each chromosome (Fig. 1a, b). The content of DNA transposons was slightly greater than that of the LTRs, accounting for 12.35% of the whole *S. album* genome, of which *mutators* accounted for 6.17%. The repeats in *S. yasi* were similar to those in *S. album*, with the genome possessing a total of 29.54% repetitive sequences, of which LTRs accounted for 7.72%, including 4.18% *copia* and 2.33% *gypsy*. DNA transposons in *S. yasi* accounted for 13.79% of the total genome length, with *mutator* being the most common type, accounting for 7.56% (Table 2). To investigate the genome evolution of Santalales more broadly, we used the same process to annotate the repetitive sequence content of the Santalales species *Malania oleifera*^17^ for comparison to the two sandalwoods. The repeat content in *M. oleifera* accounted for 78.50% of the whole genome, far exceeding the proportion of the two sandalwood species analyzed herein (Supplementary Table 4).

**Table 2:** Repeat sequence prediction in *S. album* and *S. yasi*.

		*S. album*			*S. yasi*		
Class		Count	bpMasked	%masked	Count	bpMasked	%masked
LTR	Copia	15,248	13,999,700	6.10%	12,229	9,724,920	4.18%
	Gypsy	3437	2,504,326	1.09%	4350	5,421,517	2.33%
	Unknown	4877	2,073,032	0.90%	6630	2,825,890	1.21%
TIR	CACTA	19,411	4,611,839	2.01%	14,472	4,156,173	1.79%
	Mutator	29,073	14,164,606	6.17%	91,838	17,595,622	7.56%
	PIF_Harbinger	3904	865,397	0.38%	5067	1,202,062	0.52%
	Tc1_Mariner	1648	500,329	0.22%	2691	692,298	0.30%
	hAT	5993	1,924,242	0.84%	3592	1,285,119	0.55%
	polinton	20	9220	0.00%	0	0	0.00%
nonLTR	LINE_element	1228	744,940	0.32%	638	321,391	0.14%
	Unknown	77	64,081	0.03%	0	0	0.00%
nonTIR	helitron	22,486	6,267,979	2.73%	27,394	7,137,573	3.07%
repeat_region		43,561	18,685,405	8.14%	32,039	18,360,084	7.89%
Total		150,963	66,415,096	28.93%	200,940	68,722,649	29.54%
LAI		30.81			16.68		

To annotate the genes in the *S. album* and *S. yasi* genomes, a combination of de novo, homology, and transcriptome-based annotation approaches were used to predict the gene structure in each of the two genomes. Using protein sequences from six species as a homologous database and transcriptome data from six tissues, we predicted 21,673 genes in the *S. album* genome with an average length and number of cds of 1333 bp and 5.66, respectively. The *S. yasi* genome was found to contain 22,816 genes using the same process of annotation as for *S. album*. For *S. yasi*, the average gene length and cds number were 1299 bp and 5.26, respectively (Supplementary Table 5, Supplementary Fig. 4). The BUSCO integrity evaluation of genes in *S. album* was 93.6% and 94.3% in *S. yasi* (Supplementary Fig. 2). The distribution of genes in the two genomes was generally uniform, with gene density decreasing in regions with increased LTR density (Fig. 1). In addition, we also performed functional analysis on the annotated genes. Annotations of GO (Gene Ontology) and Pfam were based on the results of InterProScan. We used eggNOG-mapper2 for synthetical annotations. After collation, 95.05% of the genes could be identified in the NR database, with 70.51% of the genes recognized by SwissProt, as well as 35.50%, 61.50%, and 93.03% by KEGG, GO, and Pfam, respectively. In total, 95.21% of genes were annotated in *S. album*. For *S. yasi*, 33.25%, 66.61%, 57.10%, 89.28%, and 91.06% of functional genes were annotated by the KEGG, SwissProt, GO, Pfam, and NR databases, respectively, resulting in 91.19% of all genes receiving functional annotations (Supplementary Table 6). In addition, 614 transfer RNA, 7064 ribosomal RNA (rRNA), 329 small nuclear RNA (snRNA), and 71 microRNA (miRNA) genes were predicted in *S. album*, and 641 transfer RNA, 5973 ribosomal RNA (rRNA), 346 small nuclear RNA (snRNA), and 76 microRNA (miRNA) genes were predicted in *S. yasi* (Supplementary Table 7).

### Gene families and phylogeny

**Fig. 2:**
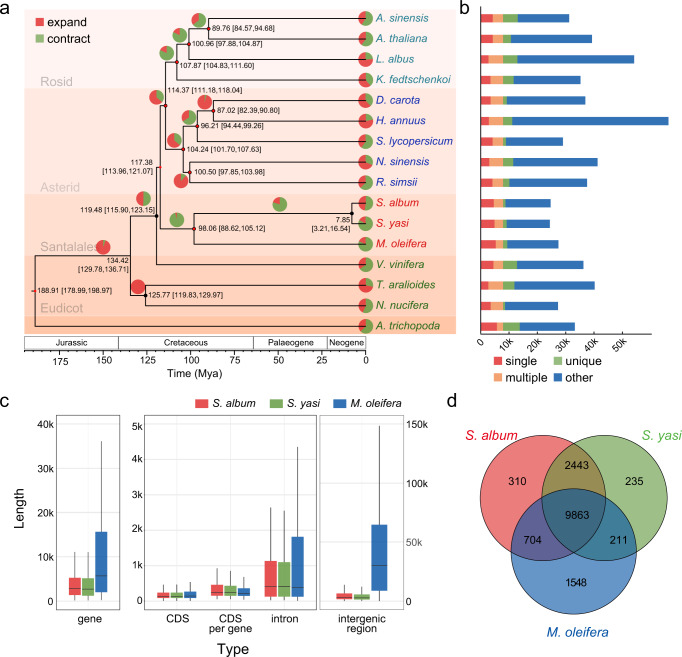
Gene families and evolution in *S. album* and *S. yasi*. **a** Phylogenetic tree of 16 species including divergence times based on 305 single-copy nuclear genes. Divergence times are indicated by the numbers at each node with 95% highest posterior density. Names in light blue indicate rosid species, names in dark blue indicate asterid species, names in red indicate Santalales, and names in green indicate early diverging eudicot lineages. Pie charts on branches represent gene families that have expanded (red) and contracted (green). Black dots at nodes indicate branch support values greater than 99, and the red dot represents a time calibration point. **b** Gene family classifications: single indicates gene families contained in all species and containing only a single-copy gene in each family; multiple indicates gene families contained in all species with multiple gene copies in each family; unique indicates genes which were not clustered; and other indicates genes that were not classified in any of the above groupings. **c** Length of gene, CDS, CDS per gene, intron, and intergenic regions in *S. album*, *S. yasi*, and *M. oleifera*. **d** A Venn diagram of shared and unique gene families in the three Santalales genomes.

A total of 30,081 gene families were obtained through the identification of homologous genes and cluster analyses of gene families using the two sandalwood species sequenced here, 13 core eudicot species and *Amborella trichopoda* as the outgroup, of which 305 were single-copy families (Supplementary Data 1). To confirm the phylogenetic position of sandalwood and the related divergence times with other lineages, we constructed a high-confidence phylogenetic tree with these single-copy families (Fig. 2a). From these results, the Superasterids and Superrosids resolved as sister clades, with Santalales diverging early from the ancestors of Superasterids and Superrosids at ~117.38 million years ago (113.96–121.07 Mya). The two sandalwood species were inferred to have diverged from a common ancestor at ~7.85 Mya (3.21–16.54 Mya) (Fig. 2a). The number of genes in the *Santalum* genomes were similar to that in *M. oleifera* (Fig. 2b). The average length of genes in *M. oleifera* was longer than those in the two sandalwood species in which the average length of CDSs were similar across these three species while the intron and intergenic regions in *M. oleifera* were much longer than those in the *Santalum* genomes (Fig. 2c). Among the three Santalales species, 15,314 gene families were found, of which *S. album* had 310 and *S. yasi* had 235 specific gene families, while *M. oleifera* had 1548 specific gene families (Fig. 2d). Functional annotations indicated that the *S. album*- and *S. yasi*-specific genes were related to “reverse transcriptase” or “gag-polypeptide of LTR *copia*-type”, suggesting that those retrotransposons recently became active, and could account for the differences in genome size between *Santalum* and *Malania*^18^ (Supplementary Table 8).

The expansion and contraction of gene families play an important role in plant diversification during evolution^19^. To gain insight into the specific differences in the sandalwood genomes and their potential biological significance, we compared gene families from *S. album*, *S. yasi*, and 11 eudicot species (Fig. 2a). It was found that *S. album* had 70 expanded and 79 contracted (*P* < 0.05) gene families, while *S. yasi* possessed 55 expanded and 174 contracted (*P* < 0.05) gene families. KEGG enrichment analyses showed that expanded genes in *S. album* and *S. yasi* were enriched in similar pathways, such as “MAPK signaling pathway”, “alpha-Linolenic acid metabolism”, “Pentose and glucuronate interconversions”, “Phenylpropanoid biosynthesis”. The contracted gene families enrichment in *S. album* and *S. yasi* were generally different. For example, “Monoterpenoid biosynthesis” was among the contracted gene families in *S. album* but was among the expanded gene families in *S. yasi*, as was the case with the “Diterpenoid biosynthesis” pathway. Genes enriched in “Enzymes with EC numbers” pathway were in the expanded gene families in *S. album* and in contracted gene families in *S. yasi* (Supplementary Table 9). What is noteworthy is that 601 of the 1548 specific gene families in *M. oleifera* were in the contracted gene families after divergence from the common ancestor of sandalwoods and *M. oleifera*. KEGG enrichment showed that these genes were mainly enriched in “Metabolism” and “Brite Hierarchies”, such as “Protein families: metabolism”, “Energy metabolism”, “Transporters”, etc. (Supplementary Table 10).

### Whole-genome triplication (WGT)

**Fig. 3:**
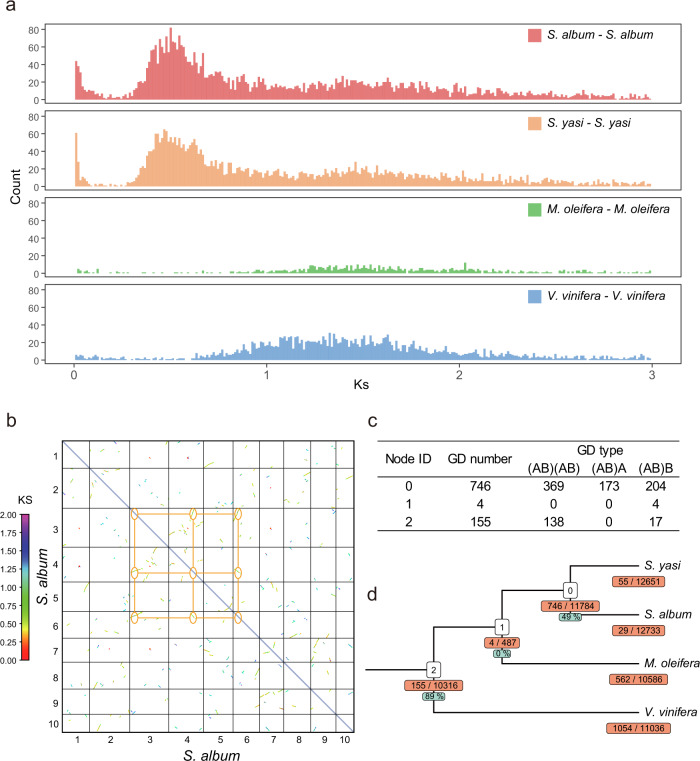
Inference of WGT events in the ancestors of *S. album* and *S. yasi*. **a** Distribution of Ks counts for paralogous genes; **b** Dot plot of DNA sequence alignments across the 10 chromosomes of *S. album*. Points representing homologous pair locations were colored according to the Ks color scale, and the area delimited in the orange boxes plot the proportional relationship of homologous pairs within a specific region. **c** Table of gene duplicate types used to infer WGTs; and **d** detection of whole-genome duplication based on the phylogenomic tree-based method, numbers in the orange box below each node is the number of duplicated gene families/total gene families, with the corresponding green box giving the percentage of (AB)(AB) type duplicates.

Detection of whole-genome triplication (WGT) events was conducted by using MCScanX to find collinear genes from three Santalales species and *Vitis vinifera* and the calculation of Ks values between these collinear genes (Supplementary Data 2–5). The Ks density map showed a shared peak (Ks 1.3–1.5) in all four species, which reflects t the γ event in the early evolutionary history of eudicots, and another clear peak (Ks 0.4–0.6), suggesting a more recent polyploidy event in the two sandalwood genomes (Fig. 3a). Previous research shows that *M. oleifera* probably did not experience a polyploidy event after the divergence of eudicots^17^, indicating that the more recent polyploidy event occurred after the divergence of *M. oleifera* and *Santalum* from a common ancestor.

Analysis of the collinearity between *S. album*, *S. yasi, M. oleifera*, and *V. vinifera* provided further strong support for polyploidy. Repeated alignment of many small fragments in the intragenomic collinearity in *S. album* and *S. yasi* resolved a WGT event in the *Santalum* lineage with more than seven colinear blocks comprising at least 15 genes of the two genomes present in triplicate with a 0.3–1 Ks, which was consistent with the results of Ks analysis (Fig. 3b, Supplementary Fig. 5). We used the collinear alignment of *V. vinifera* which shares only one ancient WGT event with all core dicots to our *Santalum* genomes as a guide (Supplementary Fig. 6). Although several blocks are missing a small part of the region, most of them in *V. vinifera* have three distinct duplications in both *S. album* and *S. yasi*, and there was a 3:1 relationship between *Santalum* and *M. oleifera*, pointing to a possible WGT event (Supplementary Fig. 7). The result of tree2GD showed a WGT event occurred after Santalales divergence but before divergence of *Santalum* (i.e., between node 0 and node 1) (Fig. 3c). In conclusion, we inferred a recent Santalaceae-specific WGT event that occurred after the divergence of Santalaceae from the ancestors of *M. oleifera*, with *M. oleifera* sharing only one ancient WGT event with all core dicots as well as *V. vinifera*.

### The evolution of a compact genome

Plant genomes have varied greatly in size during the course of evolution^20^. The two *Santalum* genomes assembled in this study were 229 Mb for *S. album* and 233 Mb for *S. yasi*. In addition to these genomes, the only other Santalales genome was from *M. oleifera*, which is 1509 Mb in size. To investigate the differences in the small genomes of the two *Santalum* species compared with those of the larger *M. oleifera*, we analyzed the genome structure and transposable elements (TEs) content of the three species. First, polyploidy events are known to be responsible for large changes in genome size^21^, but given the inferred *S. album*- and *S. yasi*-specific WGT event, we would expect the two *Santalum* species to have larger genomes with more genes than *M. oleifera*. However, the number of genes in the *Santalum* species was slightly lower than that of *M. oleifera* (Fig. 3a). When considering gene length, the average length of genes in *M. oleifera* was longer than that of either of the two *Santalum* species, but the average length of CDSs was similar across the three species (Fig. 2c). This pattern indicated that the differences in genome length were mainly the result of differences in intron and intergenic region lengths. When comparing the three species, *M. oleifera* possessed much longer introns and intergenic regions on average (Fig. 2c).

**Fig. 4:**
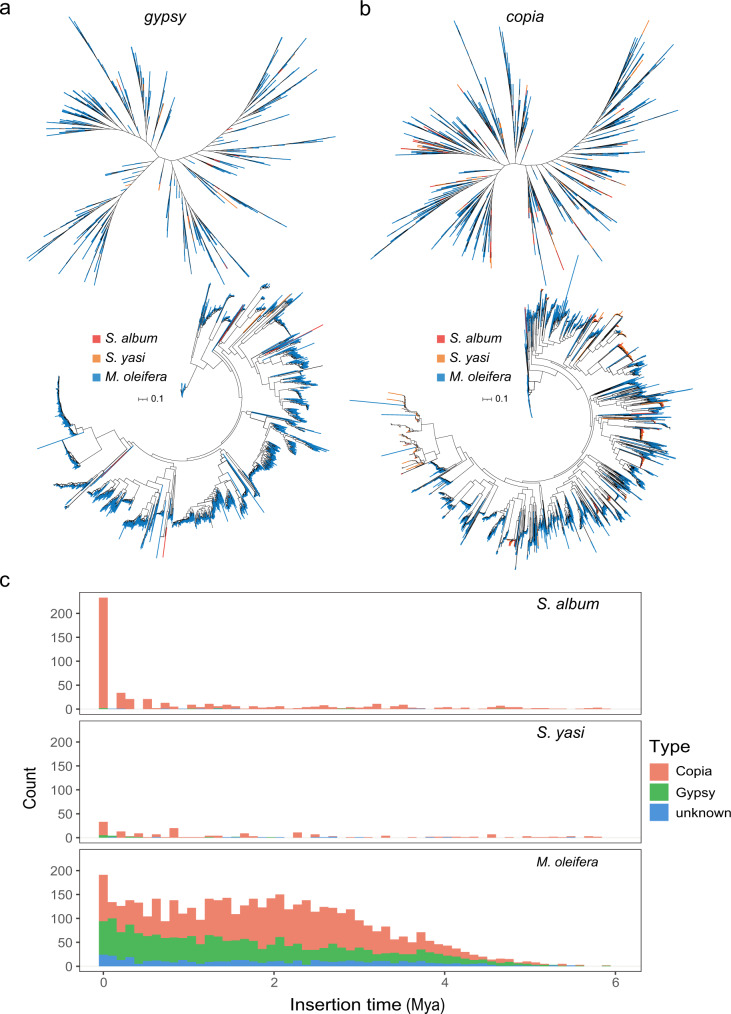
Transposable elements (TEs) analyses from three Santalales genomes. **a** Relationships of *gypsy* members using TElib (top tree) and intact LTRs (bottom tree) from *S. album* (red lines), *S. yasi* (orange lines), and *M. oleifera* (blue lines) to construct phylogenetic trees; **b** relationships of *copia* members used TElib (top tree) and intact LTRs (bottom tree) from *S. album* (red lines), *S. yasi* (orange lines) and *M. oleifera* (blue lines) to construct phylogenetic trees; **c** insertion time and copy number of *copia*, *gypsy*, and unknown LTR retrotransposons in *S. album*, *S. yasi*, and *M. oleifera*.

Specifically, within the introns and intergenic regions, we tested whether TE proliferation might underlie the differences in the genome sizes among the Santalales genomes. It was observed that repeat sequences made up the vast majority of the *M. oleifera* genome, accounting for 78.50% of the total length, while less than 30% of the *Santalum* genomes were made up of repeat sequences. Long terminal repeats (LTRs) were the most variable repeat sequences among the three species, with the fraction of LTR transposons being the highest in *M. oleifera* and the proportion of *copia* and *gypsy* families being several times higher than in sandalwood (Supplementary Table 4). Thus, we used *copia* and *gypsy* sequences of nonredundant LTR retrotransposons in the three genomes to construct phylogenetic trees and found that the two *Santalum* species contained far fewer LTR TEs than *M. oleifera*, as well as fewer LTRs in each major branch (Fig. 4a). We then used the intact *copia* and *gypsy* sequences from the three species to build trees, and the results were similar to those mentioned above (Fig. 4b).

Next, we used the complete LTR sequences of the three species to analyze the insertion time of transposons. The results showed that LTR insertion was a continuous process. However, LTR sequences of the two *Santalum* species were inferred to be relatively recent, with most of the insertions concentrated between 0 and 1 Mya, with a small number between 1 and 2 Mya and almost no insertions inferred before 2 Mya (Supplementary Data 6 and 7), while the LTR sequences of *M. oleifera* showed a large amount of accumulation from 0 to 4 Mya (Supplementary Data 8). This pattern indicated that most of the LTR TE activity in *Santalum* was recent and less abundant than that in *M. oleifera* (Fig. 4c). We counted the number and summed the length of LTR TEs in the intergenic and intronic regions of the three species and found that both the number and length of *M. oleifera* LTR TEs were greater than those of the two *Santalum* species (Supplementary Fig. 8). To assess the degree of LTR TE elimination among the three Santalales species, we calculated the ratios of solo LTRs to intact LTRs and found that the two *Santalum* species had a higher proportion of solo LTRs (Supplementary Table 11). This evidence suggests that the large differences in genome sizes within Santalales are being driven by LTR activity.

### Haustoria-specific gene expression

All species described from Santalaceae are stem or root hemiparasitic plants, with *Santalum* species being root hemiparasites^22^. To investigate gene expression in haustoria (the structures responsible for tapping into host roots) compared with ordinary root tissue, we analyzed the expression levels from different tissues of *S. album* and *S. yasi* with DESeq2. There were several differentially expressed genes (DEGs) between haustoria and roots (at corrected *P*-value (BH method) < 0.05). In *S. album*, 3608 genes were upregulated, and 4624 genes were downregulated in haustoria tissue, while there were 4930 genes upregulated and 3342 genes downregulated in *S. yasi* haustoria. The results of KEGG enrichment showed that many genes were enriched in “Carbohydrate metabolism” and “Signal transduction”. These genes were related to cell wall modification and highly expressed in the haustoria of *S. album*, such as genes coding for *pectinesterase* (*PE*), *pectin methylesterase* (*PME*), and *xyloglucan endotransglucosylase/hydrolases* (*XTH*s) when compared to root tissue and the other plant tissues from which RNA was extracted and analyzed for DEGs (Supplementary Table 12). These results suggested that cell wall remodeling was an important aspect of haustoria functioning and might be involved in modifying host tissues during the establishment of a hemiparasitic connection.

In addition to the upregulated cell wall-modifying genes, we also found that phytohormone-related genes were enriched or upregulated in *S. yasi*, including *cytokinin dehydrogenase 3* (*CKX3*), *auxin response factor 5* (*ARF5*), and *ethylene-forming enzyme* (*EFE*) (Supplementary Table 13). These results were consistent with previous studies^23^. In addition, genes in both species related to sugar transport, electron transport, and resistance to biotic and abiotic stress were differentially upregulated in haustoria compared to roots, such as *heavy metal-associated isoprenylated plant proteins* (*HIPPs*), *S-type anion channel 1* (*SLAH1*), *DOWNY MILW RESISTANCE 6* (*DMR6*), and *Peroxidase P7*. These genes are candidates for further study across the Santalaceae regarding the evolution of hemiparasitism.

### Biosynthesis of santalol in *S. album* and *S. yasi*

The two main aroma components in sandalwood essential oil are α-santalol and β-santalol^9^. The key enzymes involved in the biosynthetic pathway of α-/β-santalol have been described for *S. album*^9,24,25^. First, FPP mainly produced by the MVA pathway is converted by *SaSSy* into a blend of α-, β-, and EpI-β-sandalene, and then hydroxylated to α-, β-, and EpI-β-santalol with the involvement of *CYP736A167* and *NADPH-dependent Cytochrome P450 reductase* (*CPR*).

Based on homology search and genome functional annotation, we identified *SaSSY*, *CYP736A167*, *CPR*, and the key genes in the MVA pathway. We outlined the putative santalol biosynthesis pathway based on previously published studies. According to functional annotation, we identified *AACT*, *HMGS*, *HMGR*, *MVK*, *PMK*, *MPDC, IPPI, FPS*, *SaSSY*, *CYP736A167*, and *CPR* genes in both sandalwood species. We found that in both *S. album* and *S. yasi*, genes in the MVA pathway and the two *CPR* genes were expressed in all tissues, while *SaSSY* and *CYP736A167* showed tissue-specific expression. The two *SaSSY* genes were only found to be expressed in haustoria, stem, and roots. The overall expression pattern in *S. yasi* was slightly different from that in *S. album*. One notable difference was that only one of the two *SaSSY* in *S. yasi* was highly expressed in roots and haustorium, while both the two *SaSSY* genes had high expressions only in root in *S. album*. In *S. album*, the expression of *CYP736A167* in root was higher than that in haustorium, and the opposite was true in *S. yasi* (Supplementary Fig. 9). The results of our study differ from those of previous studies, which may be related to the different growth stages of the plant samples, and will require further study to elucidate how the santalol pathway differs among tissues.

## Discussion

In this study, we assembled chromosome-level genomes for two sandalwood species. Both *S. album* and *S. yasi* were assembled into 10 pseudochromosomes and had a length of 229.59 Mb and 232.64 Mb, respectively. The identified repeats accounted for 28.93% and 29.54% of the genome, and 21,673 and 22,816 genes were predicted from *S. album* and *S. yasi*, respectively. The identification of gene families, phylogenetic relationships, and gene family contraction and expansion of the two *Santalum* species were also analyzed. We compared differentially expressed genes in the roots and haustoria and found that several DEGs related to cell wall modification and phytohormone regulation were upregulated in the haustoria. We also classified the main genes involved with santalol biosynthesis and mapped their expression patterns to different tissues where the key *SaSSY* gene was found to be highly expressed in the haustoria and root.

The accurate genome assembly of highly repetitive GC-rich chromosomal regions has long presented a challenge over conserved gene-rich regions^26,27^. The circos plots in Fig. 1 showed that some regions of the genomes contained higher levels of LTRs, and GC percentages compared to adjacent stretches. Our detailed analysis found that although the depth is generally lower in all these areas with high GC content, they are supported by NGS and HiFi reads. Some of these regions were enriched with ncRNA (i.e., mainly rRNA) genes, such as the 10,875,000–12,500,000 bp and 18,700,000–19,925,000 bp regions in Chr1 of *S. album* (Supplementary Fig. 10a). All GC-rich regions in Chr1 of *S. album* and Chr1 and Chr2 of *S. yasi* were found to have a high density of TIR mutator repeats which was different from that the high GC content region in Chr10 of *S. yasi* (Supplementary Fig. 10b, c, d). Since these positions are located in the middle of the chromosomes, which are expected to contain high repeat abundance, detailed follow-up work is needed to confirm that these regions are accurately assembled and treated as real centromeric regions.

The placement of Santalales in the eudicot tree of life has long been problematic, and the position resolved here using 305 single-copy genes is unlikely to silence debate on this topic. It should be mentioned that while our genomic sampling is extensive, the key samples from lineages such as Dilleniales, Berberidopsidales, and Caryophyllales also need to be sampled to assess more comprehensively the placement of early diverging branches. In APG IV, the Santalales was resolved in an early diverging position to Superasterids, while in our analysis, the Santalales branch was diverging early to a clade of Superasterids+Superrosids^28^. Cases of alternative resolutions are common among studies attempting to resolve the branching pattern of early diverging lineages of eudicots when different molecular datasets and sampling strategies were applied^29^. For instance, a genomic study using a similar approach of extracting single-copy genes from genomes but with a more exhaustive phylogenetic approach than ours resolved alternative topologies from those previously proposed in APG IV^30^. A recent study of the *M. oleifera* genome placed it in a clade with *V. vinifera* and sister to the rosid clade using an ML method and 282 single-copy genes, which also differs from the APG IV topology^17^. As more genomes are sequenced, the resolution of these early diverging lineages is likely to improve our understanding of the evolutionary variations in those species.

In addition to the differences between datasets and phylogenomic methods producing conflicting topologies, biological factors such as WGD/WGT can disrupt previously strong phylogenetic signals^31^. In our collinear alignment analysis of three Santalales species and *V. vinifera*, we concluded that *M. oleifera* only possessed one ancient WGT event shared with all core eudicots, whereas in the two *Santalum* genomes, we found evidence for a more recent WGT event after differentiation from the ancestors of *M. oleifera*. From this inference, the expected ratios of the orthologous genes of sandalwood with *M. oleifera*/*V. vinifera* and *M. oleifera* with *V. vinifera* should be 9:3 and 3:3, but the observed ratios were 3:1, 2:2, or 2:1. Also, there was a high degree of collinearity between *S. album* and *S. yasi* in both genes and genome structure (Supplementary Figs. 11 and 12). We also found some 1:2, 2:2, 1:3, 2:3, and 3:3 relationships between *S. album* and *S. yasi* in the collinear dot plot. It is well known that subfunctionalization of genes, pseudogeneization, rapid gene differentiation, and diploidization occur at higher frequencies after duplication events, in which sequence variation, chromosomal rearrangements, divisions, fusions, and other molecular variations take place^32^. These events lead to changes in genome structure that may eventually lead to gene loss, whereby only a fraction of the WGD/WGT-replicated genes remain, and the phylogenetic signals can be convoluted. Thus, very ancient WGD/WGT events often do not result in discernable collinear blocks in extant species. The abnormal proportions with *V. vinifera* may be the result of homologous gene pair loss through the long evolutionary history of these lineages. Regardless, it is clear that different evolutionary trajectories followed between the *Santalum* and *Malania* lineages, given their vast differences in genome size and TE content.

We found that the genome size of *M. oleifera* was over six times that of either of the *Santalum* genomes. Analyses indicated that the *Santalum*-specific WGT event did not explain the difference in genome sizes between the two Santalales lineages but that the proliferation and retention of transposons appear to have driven genome expansion in the *Malania* lineage. Given that changes in genome size are driven both by bursts of LTR retrotransposon activity and elimination through unequal homologous recombination and double-strand break repair^33^, it is also possible that the *Malania* genomes lack efficient removal mechanisms and that *Santalum* genomes possess an abundance of such mechanisms. We found retrotransposon-related genes in expanded gene families of the two *Santalum* genomes, and the solo LTR to intact LTR ratio in them was much higher than that in *M. oleifera*, suggesting a rapid purging mechanism. We suspect that this might be one of the mechanisms by which *Santalum* genomes reduce the retention of LTR TEs and limit subsequent transposon activity.

## Methods

### Plant materials and genome sequencing

High-quality genomic DNA was extracted from leaves using a modified CTAB method^34^. Leaf material from plants was collected at the Experimental Station of the Research Institute of Tropical Forestry, Chinese Academy of Forestry, Guangzhou, China. The extracted DNA was assessed for concertation and quality using a NanoDrop 2000 spectrophotometer (NanoDrop Technologies Wilmington DE USA), Qubit 3.0 Fluorometer instrument (Life Technologies Carlsbad, CA, USA), and 0.8% agarose gel electrophoresis. We used 1 μg DNA and the MGI DNA Library Universal Kit (Novizan, Nanjing) in strict accordance with the manufacturer’s recommendations to construct the sequencing libraries. The concentration and fragment size distribution of the samples in the library were determined using a Qubit 3.0 Fluorometer (Life Technologies Carlsbad, CA, USA) and a Bioanalyzer 2100 system (Agilent Technologies, CA, USA), respectively. After the library was quantified, sequencing was performed on the MGISEQ-2000 platform (sequencing services were provided by Frasergen Bioinformatics, Wuhan)^35^. In addition, high-molecular-weight DNA was prepared and used to construct linked read libraries using a PacBio SMRTbell library from a SMRTbell Express Template Prep Kit 2.0, following the manufacturer’s protocols. After the library was constructed, it was sequenced on the PacBio Sequel II platform^36^.

### RNA sequencing and gene expression analysis tissue

Total RNA from the root, haustorium, stem, and leaf samples were isolated using a phenol/chloroform method^37^, and the purity and integrity were checked before RNA-Seq library construction. For each tissue type, three biological replicates under the same condition were taken. All sequencing libraries from all samples of the four tissues were constructed by mRNA-seq prep kit and sequenced on an MGISEQ-2000 using the PE150 mode.

We used HISAT2^38^ to map the RNA-seq data to the genome. Read counts of these samples were calculated using featureCounts^39^, and PCA was used to verify the correlation among samples (Supplementary Fig. 13). Differential gene expression analysis was conducted using the counts of three biological replicates with R package DESeq2 within each tissue (at corrected *P*-value (BH method) < 0.05).

### Hi-C library construction and sequencing

The Hi-C library was constructed according to standard procedures. First, formaldehyde was used to fix the leaves of *S. album* and *S. yasi* and crosslink DNA. Second, sticky ends were produced by treating the crosslinked DNA with the restriction enzyme MboI. Third, biotin-labeled nucleotides were introduced by terminal DNA repair to enable subsequent DNA purification and capture. Next, we used cyclization of the end-repaired DNA and DNA fragments to ensure the location of the interacting DNA. Finally, we isolated, purified, and assessed the DNA samples; thereafter, the biotinylated DNA fragments were enriched and sheared to a fragment size of 100–500 bp to construct a sequencing library. After A-tailing, pulldown, and adapter ligation, the DNA library was sequenced on an MGISEQ-2000 in PE150 mode. Juicer v1.6.2 (https://github.com/aidenlab/juicer) was used to obtain valid read pairs.

### Estimation of genome size, heterozygosity, and repeat content

Genomescope2 was used to estimate the genome size, heterozygosity, and repeat content of *S. album* and *S. yasi* with a 21-mer frequency from data generated from clean paired-end short reads with default parameters^40^. The properties of the *S. album* and *S. yasi* genomes can be reflected in the distribution of 21-mers following a Poisson distribution.

### Sequence assembly and quality evaluation

HiFi read assessment and de novo assembly were performed using Hifiasm (0.14-r312). For Hi-C sequence data, Bowtie2 (v2.3.2) was used to filter out low-quality and unvalidated paired-end reads. Valid read pairs were used to construct interaction matrices to obtain chromosome-scale scaffolds by juicer. Purge_dups (https://github.com/dfguan/purge_dups) was used to find redundant contigs and remove them selectively, resulting in a nonredundant draft genome. Using short reads with BWA (v0.7.9a) (https://github.com/lh3/bwa) and Pilon (v1.22), the draft genome was subjected to a final round of base-error correction (polishing)^41,42^. Using BUSCO (v4.0.1) and the embryophyta_odb10 databases (issued 2020-08-05, including 1614 proteins), the completeness of the assembled *S. album* and *S. yasi* genome sequences was analyzed^43^. To measure genome coverage based on read mapping rates, NGS short reads and CLR subreads were mapped against the assembled genome sequences. The distribution of GC content was used to detect sample contamination.

### Genome annotation

The EDTA (v1.9.4) pipeline was used to identify *S. album* and *S. yasi* repeat sequences^44^. Several programs, including LTR_FINDER, LTRharvest, LTR_retriever, Generic Repeat Finder, HelitronScanner, TIR-Learner, RepeatMasker, and RepeatModeler, as well as a series of integration scripts, were used in this method to annotate and identify LTR, LINE, SINE, Helitron, MITE, and other retrotransposon and transposon sequences more accurately and completely. RepeatMasker (v4.1.1)^45^ and RepeatModeler (v2.0.1)^46^ were used separately to assist in verifying the results of EDTA pipeline (Supplementary Table 14). In addition, we utilized Tandem Repeat Finder (http://tandem.bu.edu/trf/trf.html) to independently predict tandem repeats in the genome^47^. We used CMSScan (https://github.com/ajinabraham/CMSScan) to mine ncRNA information with the Rfam nonredundant database, which is based on the homology annotation of ncRNAs, including tRNA, rRNA, miRNA, and snRNA^48^.

To annotate the gene structure, we used three methods: homology, de novo, and transcript-based annotation. For homology annotation, we used the published protein information of *Nelumbo nucifera*, *Kalanchoe fedtschenkoi*, *V. vinifera*, *Arabidopsis thaliana*, *Malania oleifera*, *Beta vulgaris*, and *Solanum lycopersicum* as references and predicted their gene structure with genewise after tblastn alignments. We assembled transcripts using the de novo mode in Trinity (v2.6.6)^49^. Then, we used HISAT2 (v2.1.0) to map the RNA-seq data to the genome and obtained genome-guided transcripts by StringTie (v2.1.4) and TransDecoder (v5.5.0)^50–52^. AugustUS (v3.3.3), GlimmerHMM (v3.0.4), and snap (v2006-07-28) were used in the de novo annotations^53,54^. Finally, we used EVidenceModeler (v1.1.1) for integration^55^.

To annotate the functions of all predicted genes, we used blastp (BLAST 2.11.0 + ) to align the protein sequences of *S. album* and *S. yasi* to the public databases Swiss-Prot (https://ftp.uniprot.org/pub/databases/uniprot/current_release/knowledgebase/complete/uniprot_sprot.fasta.gz) and NR (https://ftp.ncbi.nlm.nih.gov/blast/db/FASTA/nr.gz). From these, we created functional assignments based on the best hits. The InterPro database was used to identify protein domains, including GO and Pfam annotations^56^. Additionally, the KAAS server (https://www.genome.jp/kegg/kaas) was used to find the KEGG pathways.

### Comparative phylogenomics

Gene families were clustered using protein sequences by the longest transcript from *N. nucifera*, *Trochodendron aralioides*, *K. fedtschenkoi*, *Lupinus albus*, *Aquilaria sinensis*, *Arabidopsis thaliana*, *Malania oleifera*, *Nyssa sinensis*, *Rhododendron simsii*, *S. lycopersicum*, *Daucus carota*, and *Helianthus annuus*. With the parameter “-S diamond”, Orthofinder2 (v2.5.2) was used to classify the gene families^57^. The orthologs were aligned using MAFFT (v7.480)^58^. Based on 323 single-copy genes, a 16-species phylogenetic tree was constructed by using RAxML (v8.2.12) with “-p 12345 -m GTRGAMMA -f a -o A_ trichopoda -x 12345 -# 1000”. MCMCTree in PAML (v4.8) was employed to estimate divergence times between the sampled species with “clock = correlated rates, model = REV, burnin = 50,000, nsample = 100,000”, with calibration times obtained from the TimeTree database (http://www.timetree.org/)^59^. CAFE (v4.1) was used to detect orthologous gene family expansion and contraction^60^. We used an all-to-all search in blastp (e-value cutoff < 1e-5) to identify homologous pairs to explore the evolution of *S. album* and *S. yasi*. To detect syntenic blocks, the results were examined using MCscanX (v0.8) with “-s 15”, which sets the minimum number of genes in a colinear block to 15. YN00 in PAML was called by WGDI (v0.4.7) to calculate Ks between collinear genes in each pair of *S. album*, *S. yasi*, *M. oleifera*, and *V. vinifera* within each species and draw dotplots between *S. album* and *S. yasi* with the Ks values added. JCVI (v1.1.14) was used to draw dotplots of *S. album* to *M. oleifera*, *S. yasi* to *M. oleifera*, *S. album* to *V. vinifera*, and *S. yasi* to *V. vinifera* with default parameters.

### Verification of WGT events with tree2GD

Tree2GD (v2.5) with default parameters was used to confirm the occurrence of the WGD/WGT event in Santalales. WGD/WGT events were considered to have occurred according to any of the following conditions: (1) gene duplication (GD) > 500, of which the number of (AB)(AB) type duplication genes is over 250; (2) duplication genes > 1500, of which (AB)(AB) type duplication genes are over 100, and at the same time, the sum of (AB)(AB) type duplication genes and (AB)A or (AB)B type duplication genes are over 1000^61^.

Based on the phylogenomic Tree2GD method, the same clear WGD/WGT signal was also detected. The results of Tree2GD showed that the ancestors of *S. album* and *S. yasi* (i.e., node 0) retained 746 duplicated genes, of which 369 were (AB)(AB)-type duplicate genes and met the conditions for determining a WGD/WGT event, with one WGD/WGT event occurring after Santalales differentiation but before differentiation of *S. album* and *S. yasi* (i.e., between node 3 and node 2) (Fig. 3c, d).

### LTR-based phylogenic trees

We filtered the nonredundant LTR retrotransposons of *copia* and *gypsy* from the TE-lib in the results of the EDTA pipeline. FastTree (v2.1.10) with default parameters was used to construct the trees of *copia* and *gypsy*, respectively.

### GO and KEGG enrichment analyses

The functional annotation of protein sequences in *S. album* and *S. yasi* based on the eggNOG-mapper (v2.0.1) program was used for the GO and KEGG enrichment analyses. With the R package AnnotationForge (v1.36.0), we constructed two species-specific function databases for each species (https://bioconductor.org/packages/AnnotationForge/). The program clusterProfiler (v4.2.192) was used for the enrichment results, and we visualized it using the R package ggplot2 (https://github.com/tidyverse/ggplot2). Tbtools was used for the enrichment of haustoria-specific gene differential expression^62^.

### Statistics and reproducibility

For each tissue type, three biological replicates under the same condition were taken. Differential gene expression analysis was conducted using the counts of three biological replicates with R package DESeq2 within each tissue (at corrected *P*-value (BH method) < 0.05, log2FoldChange > 1 or log2FoldChange < −1). The final value in FPKM of the genes represents the average FPKM of the three biological replicates.

### Reporting summary

Further information on research design is available in the Nature Portfolio Reporting Summary linked to this article.

## Supplementary information

Supplementary Information

Description of Additional Supplementary Files

Supplementary Data 1-8

Reporting Summary

## Data Availability

All data supporting the results of this study are included in the manuscript and its additional files (Supplementary Table 15). The RNA-seq data from six tissues have been submitted to the CNCB (https://www.cncb.ac.cn/) under accession number PRJCA009490. The whole-genome sequence data and annotations reported in this paper have been deposited under accession number GWHCBIR00000000 which is publicly accessible at https://ngdc.cncb.ac.cn/gwh. Functional annotations and evolution results have been submitted to figshare (Peng, DAN (2023): PRJCA009490. figshare. Dataset. 10.6084/m9.figshare.22877087.v1).
