# Author Correction: Chromosome-level genome assemblies from two sandalwood species provide insights into the evolution of the Santalales

**DOI:** 10.1038/s42003-023-05062-z

**Published:** 2023-06-28

**Authors:** Zhou Hong, Dan Peng, Luke R. Tembrock, Xuezhu Liao, Daping Xu, Xiaojin Liu, Zhiqiang Wu

**Affiliations:** 1 Research Institute of Tropical Forestry, Chinese Academy of Forestry, 510520 Guangzhou, China; 2 College of Agriculture, Center for Genomics and Biotechnology, Fujian Agriculture and Forestry University, 350002 Fuzhou, China; 3 Shenzhen Branch, Guangdong Laboratory for Lingnan Modern Agriculture, Genome Analysis Laboratory of the Ministry of Agriculture, Agricultural Genomics Institute at Shenzhen, 518120 Shenzhen, China; 4 Kunpeng Institute of Modern Agriculture at Foshan, Shenzhen Branch, Guangdong Laboratory of Lingnan Modern Agriculture, Agricultural Genomics Institute at Shenzhen, Chinese Academy of Agricultural Sciences, 518124 Shenzhen, China; 5 Department of Agricultural Biology, Colorado State University, Fort Collins, CO 80523 USA

**Keywords:** Plant evolution, Phylogenetics

Correction to: *Communications Biology* 10.1038/s42003-023-04980-2, published online 01 June 2023.

In this article the author name Xiaojin Liu was incorrectly written as Xiaojing Liu. The original article has been corrected.

