# Supplementary material for: Chromosome-level genome assemblies from two sandalwood species provide insights into the evolution of the Santalales
Source: Commun Biol. 2023 Jun 1;6:587. doi: 10.1038/s42003-023-04980-2 (PMC10235099; doi:10.1038/s42003-023-04980-2)
Supplement: Supplementary file 1 — Supplementary Information [file 42003_2023_4980_MOESM1_ESM.pdf]

Supplemental Material

Supplemental Figure

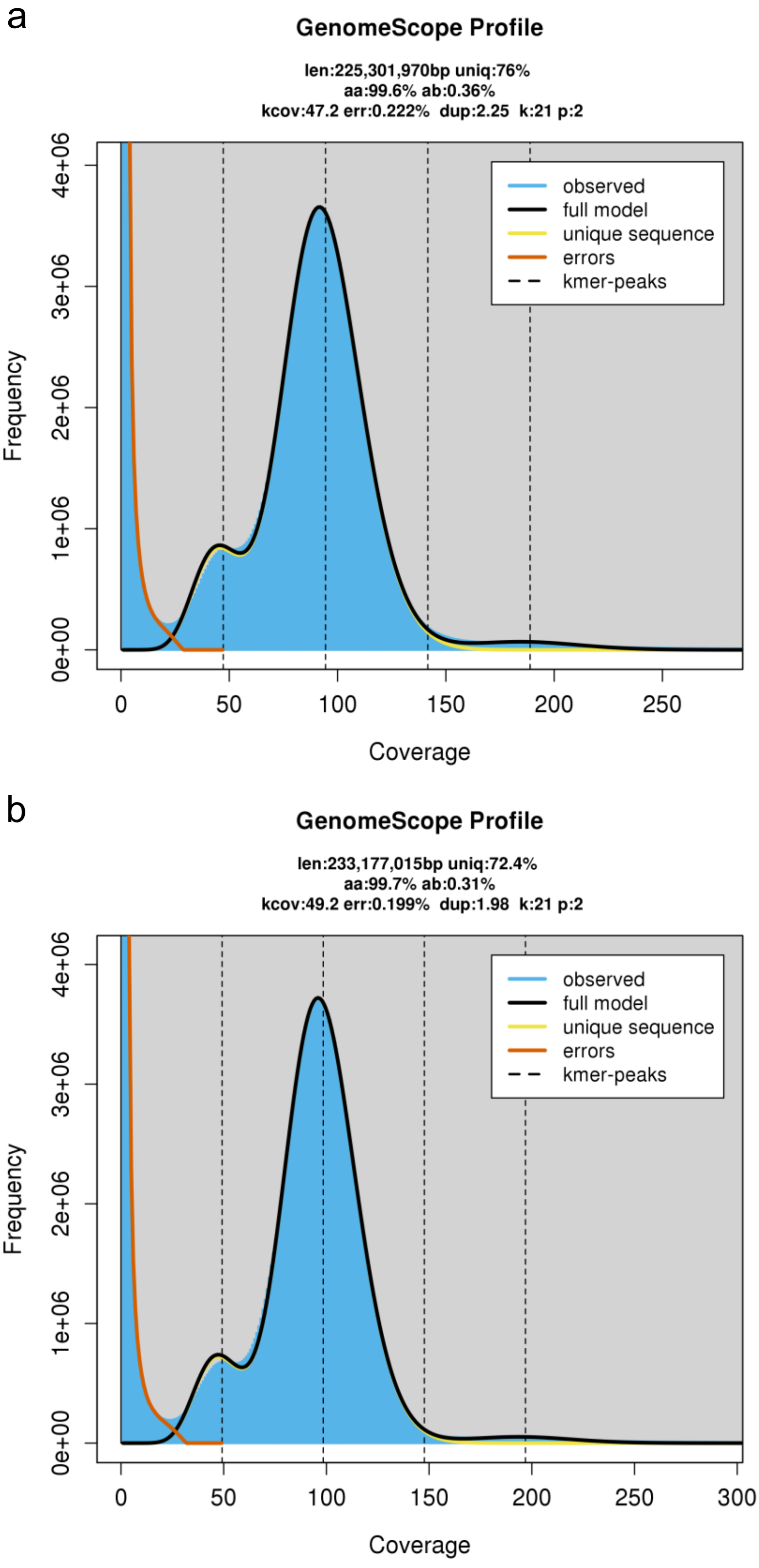

Supplementary Figure 1: K-mer analysis for estimating the genome size of *S. album* (a), and *S. yasi* (b).

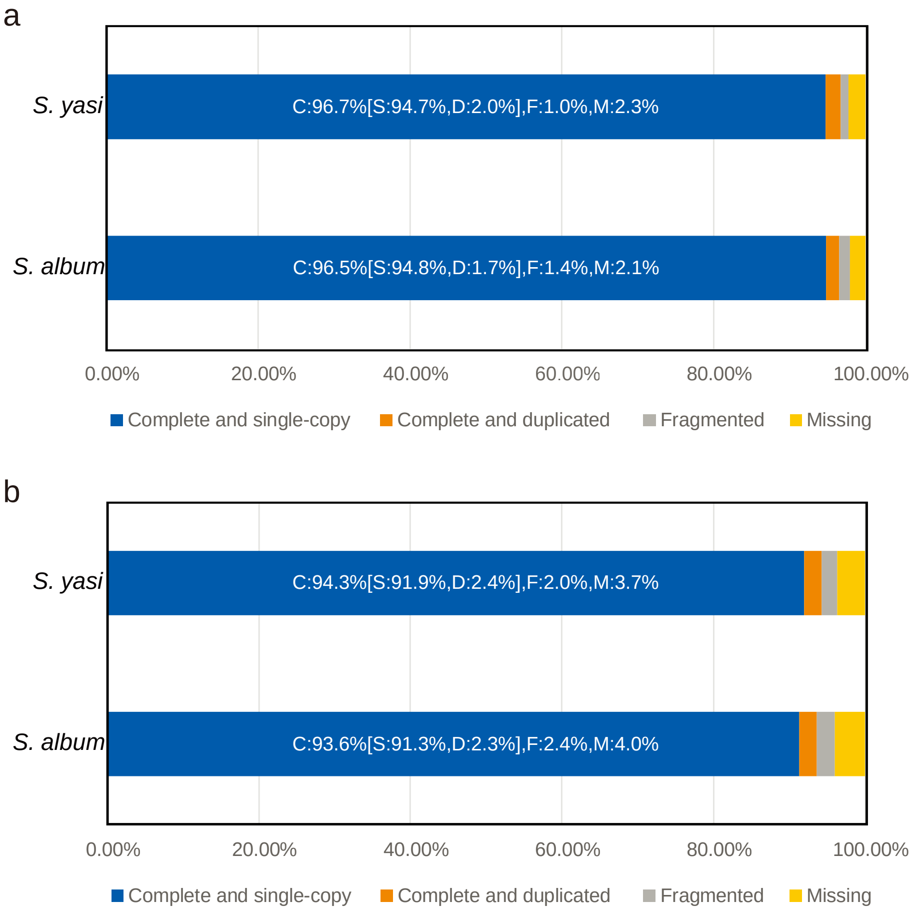

Supplementary Figure 2: BUSCO assessment results. a. BUSCO assessment of *S. album* (a), and *S. yasi* (b) genome. b. BUSCO assessment of genes in *S. album* (a), and *S. yasi* (b). Abbreviations are C: Complete BUSCOs; S: Complete and single-copy BUSCOs; D: Complete and duplicated BUSCOs; F: Fragmented BUSCOs; and M: Missing BUSCOs.

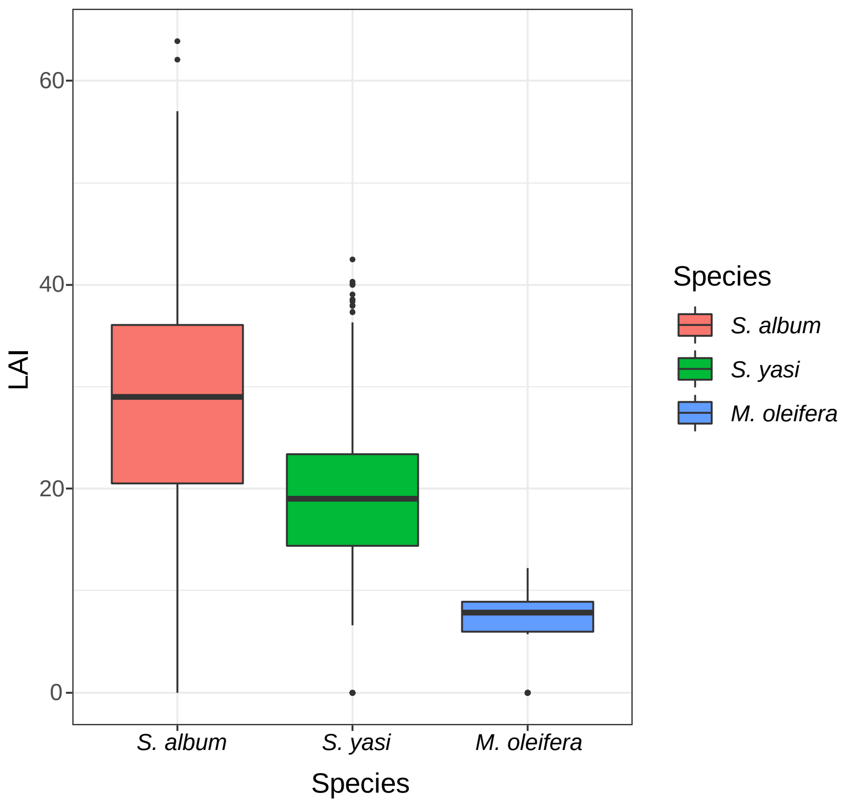

Supplementary Figure 3: LAI of *S. album*, *S. yasi*, and *M. oleifera*

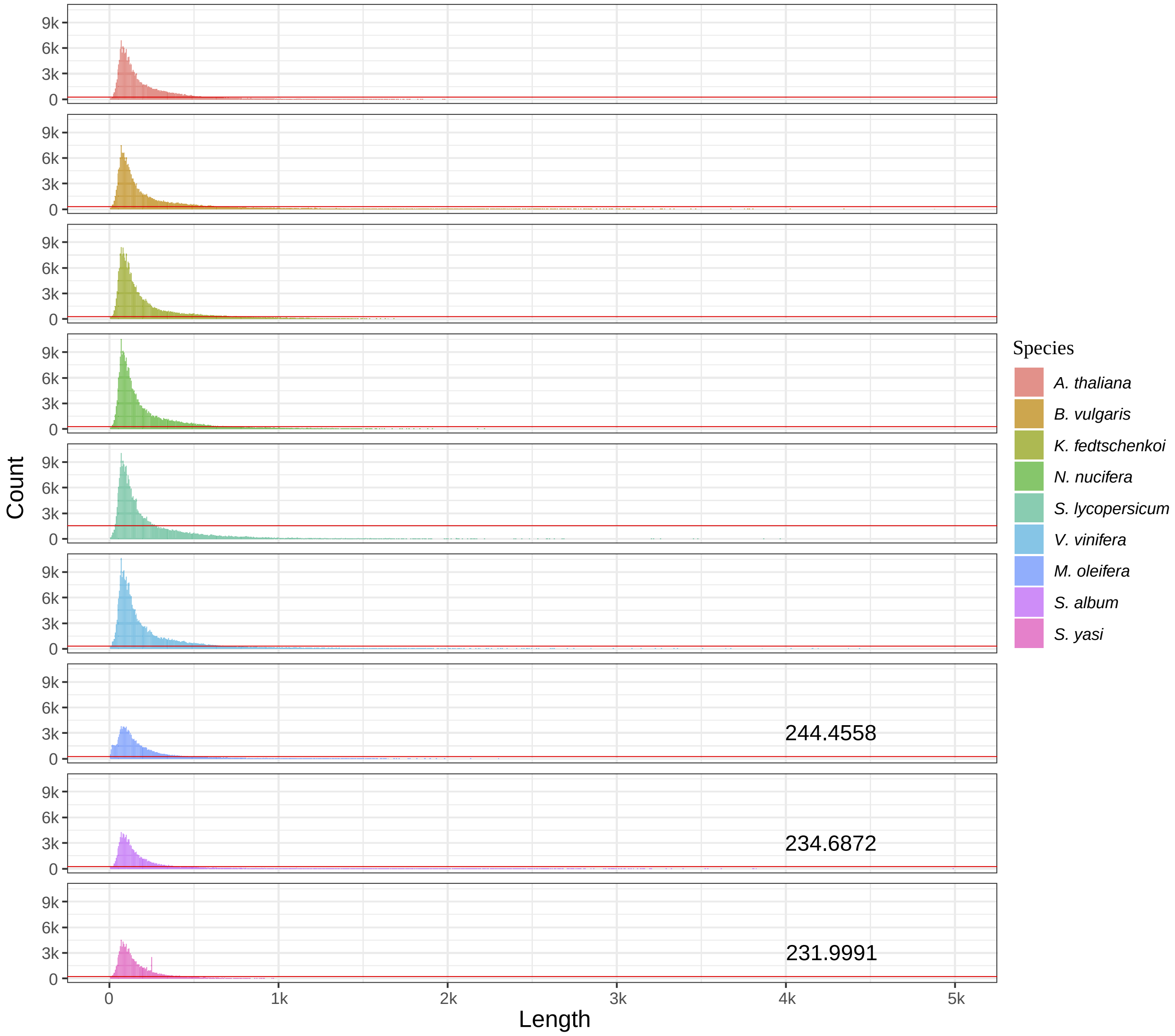

Supplementary Figure 4: Cds length counts distribution for cds lengths from *S. album*, *S. yasi* and other 7 species used in homology annotation. Red lines are the average cds length. The numbers above the red lines are the values of average cds lengths.

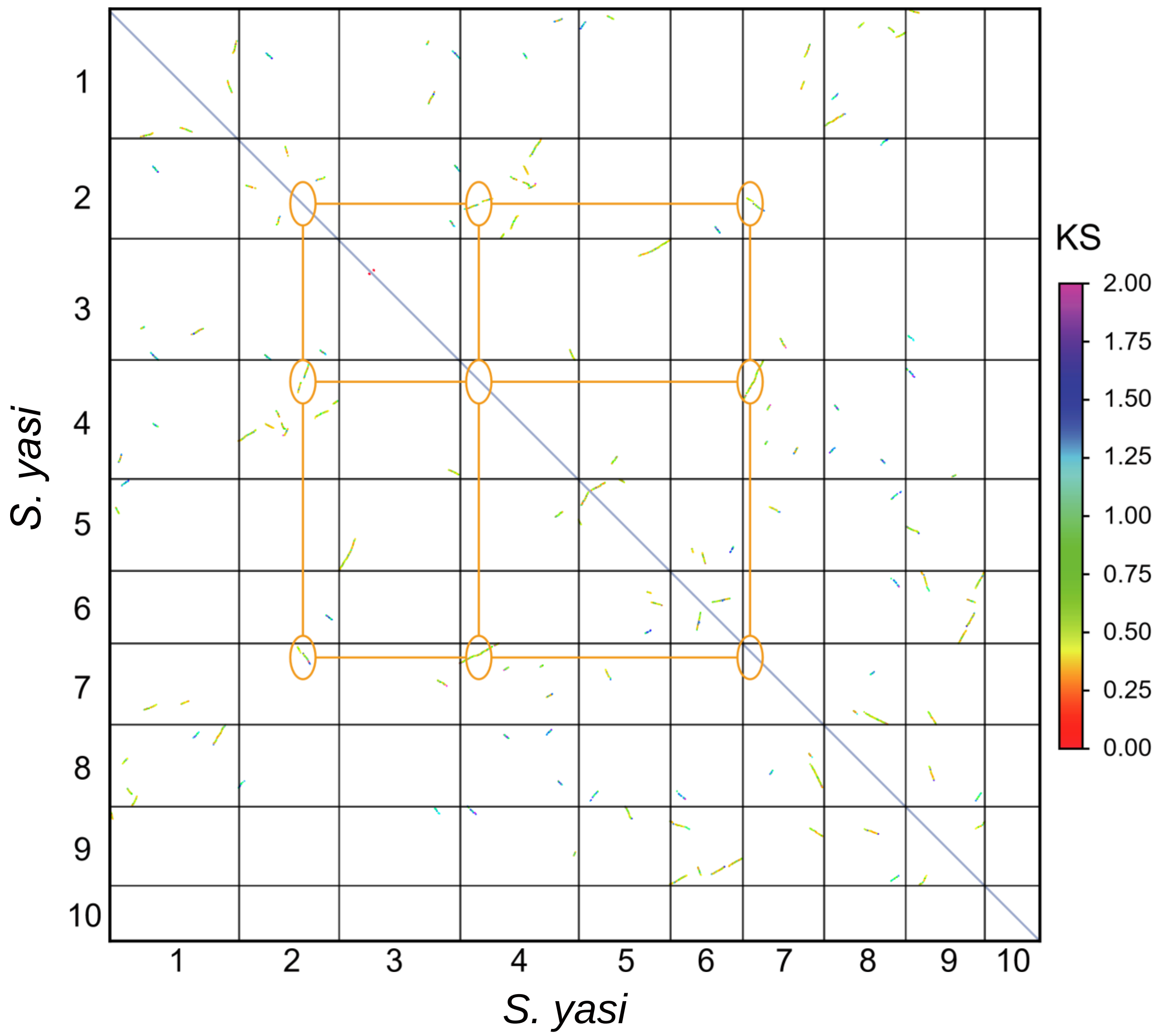

Supplementary Figure 5: Dot plot of gene alignments of *S. yasi*. Points representing homologous pair locations were colored according to the Ks color scale, and the area delimited in the orange boxes plot the proportional relationship of homologous pairs within a specific region.

a

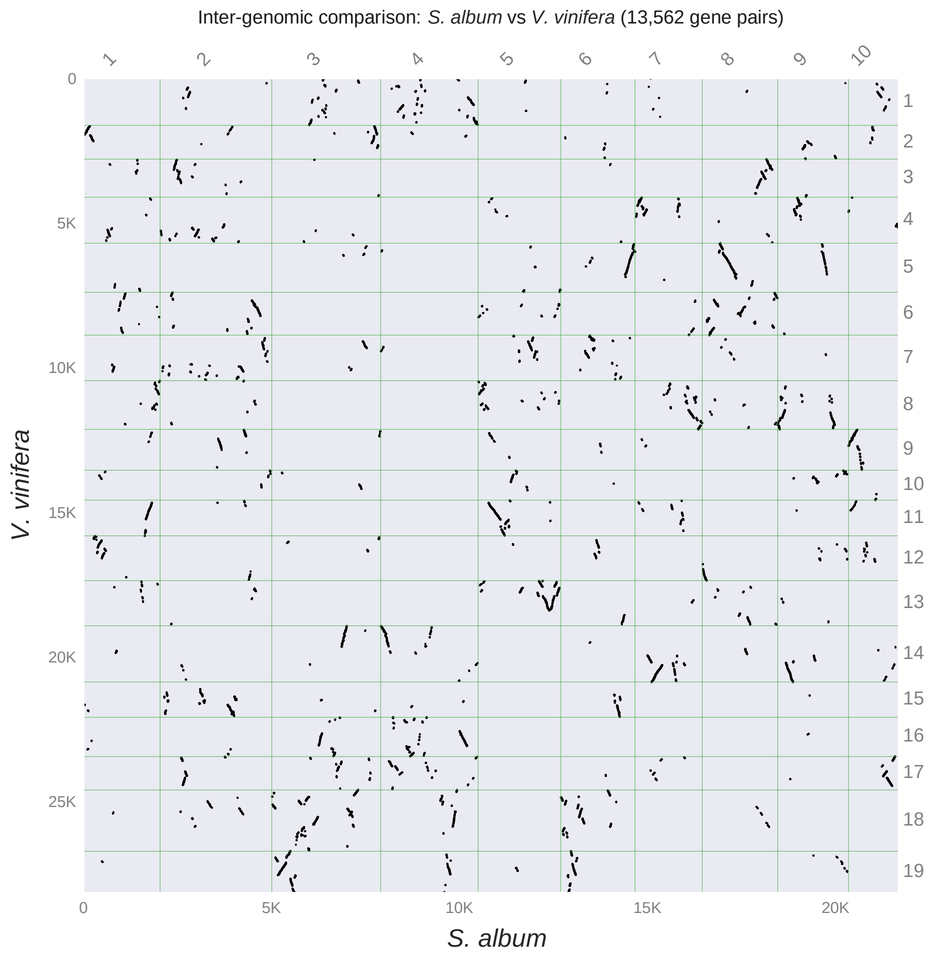

b

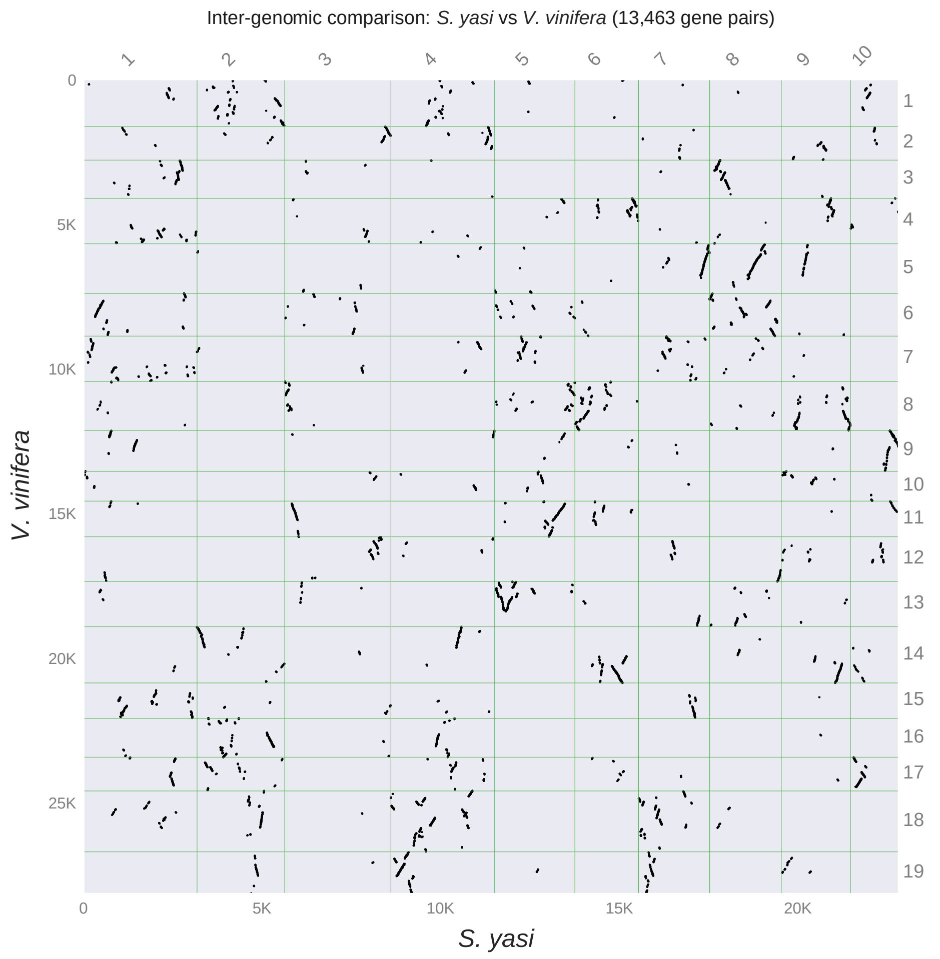

Supplementary Figure 6: Dot plot of gene alignments of *S. yasi* and *S. yasi* with *V. vinifera*.

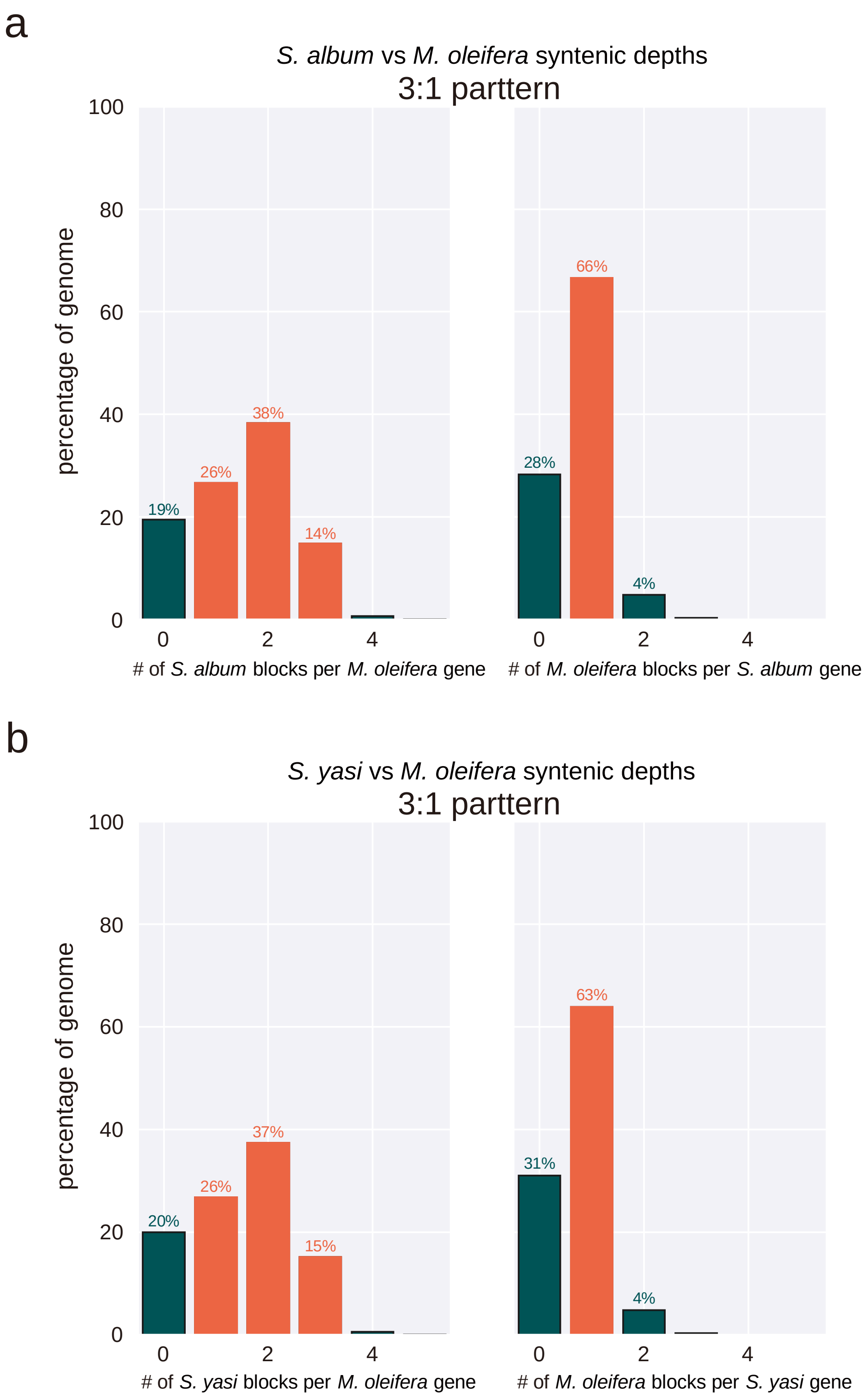

Supplementary Figure 7: patterns of Syntenic depths

Bar chart shows the ratio of the number of homologous genes in one species to the number of homologous genes in another species. The x axis shows the number of corresponding homologous genes, and the y axis shows the proportion of the genes to all genes. Orange columns indicate the most abundant types of proportions. a. The corresponding relationship between *S. album* and *M. oleifera*; b. The corresponding relationship between *S. yasi* and *M. oleifera*.

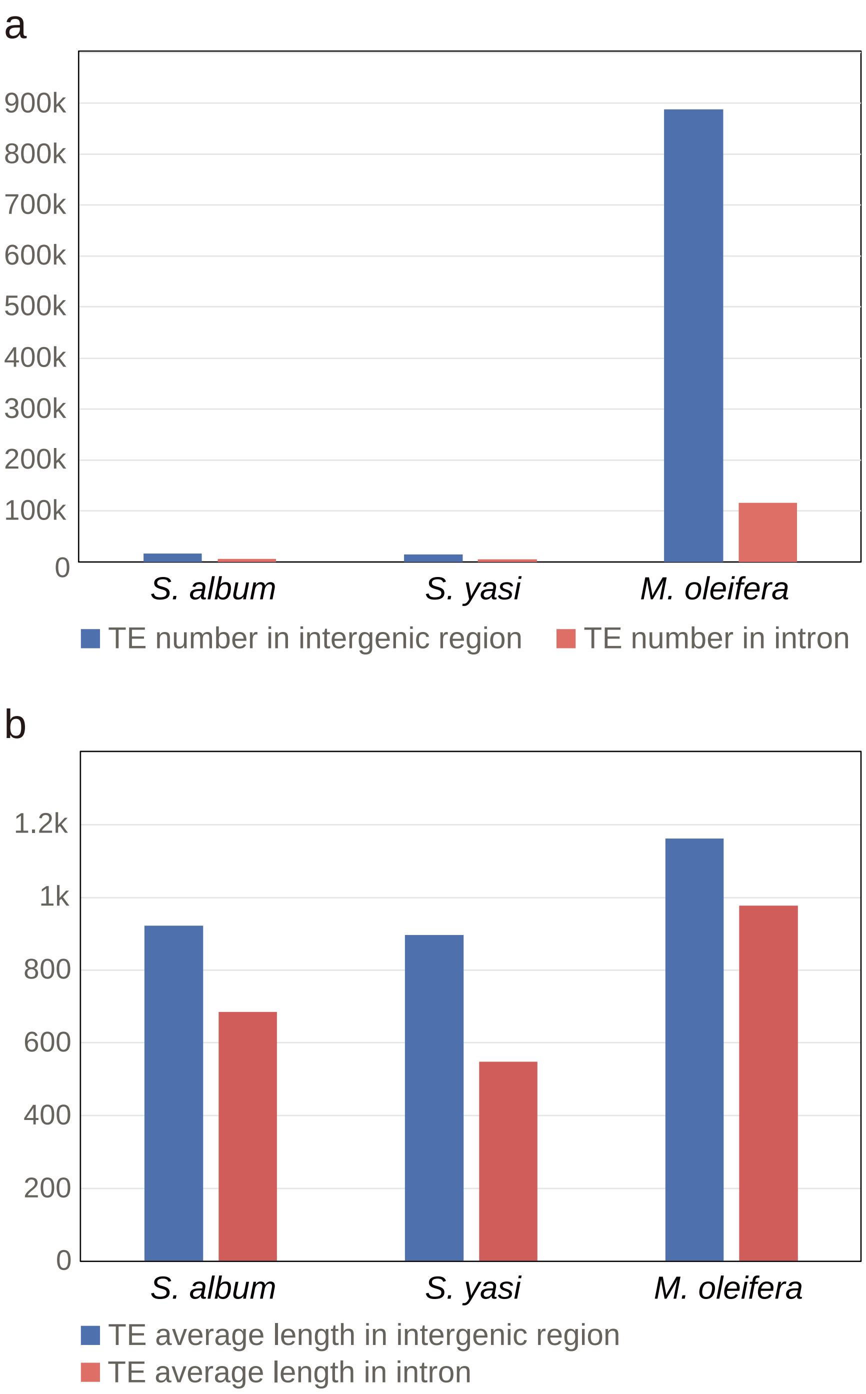

Supplementary Figure 8: Average TE number (a) and length (b) in introns and intergenic regions in *S. album*, *S. yasi,* and *M. oleifera*.

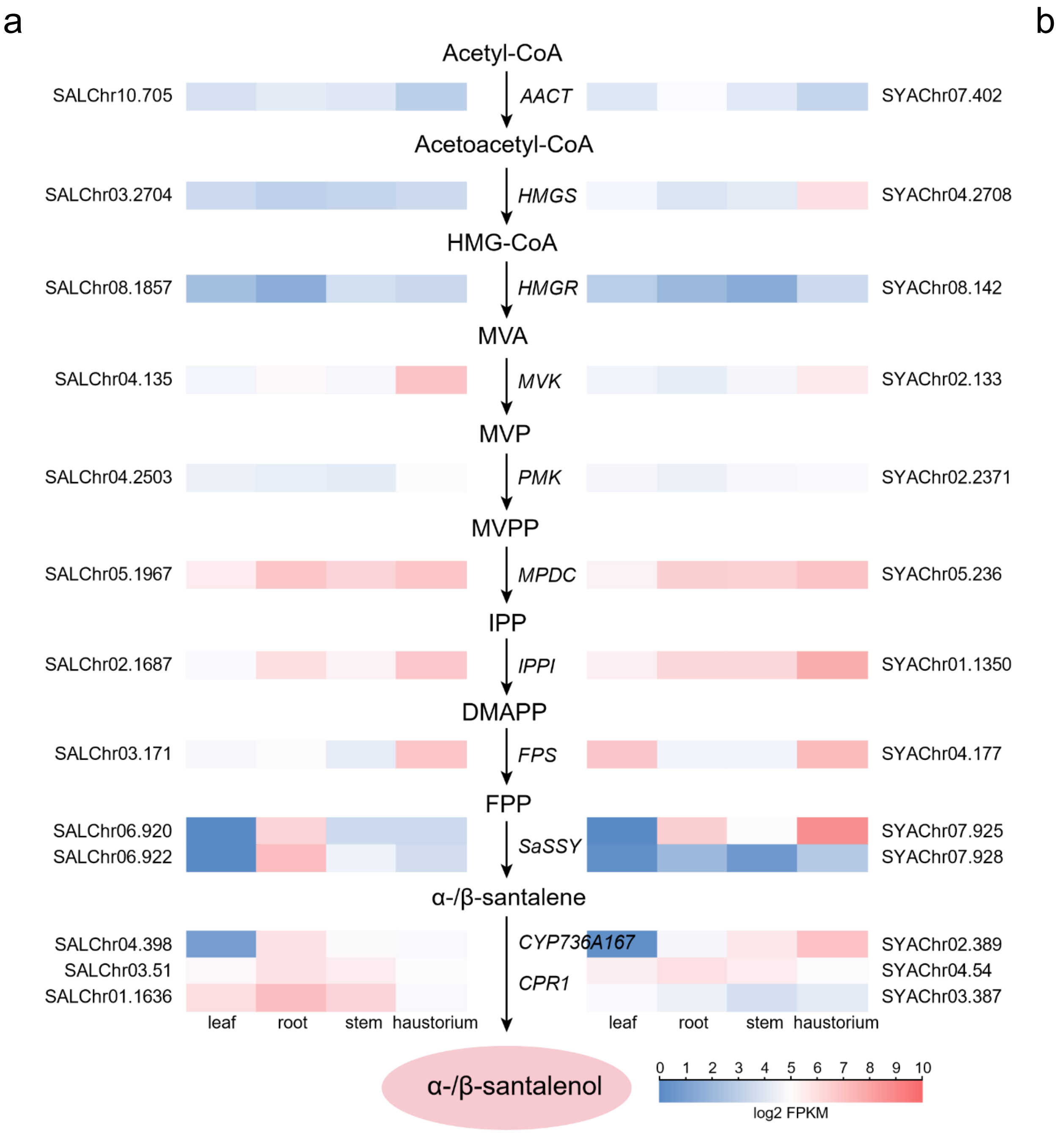

Supplementary Figure 9: Simplified scheme biosynthetic pathway of santalol synthesis in *S. album* (a), and *S. yasi* (b). *AACT*, *acetoacetyl-CoA thiolase*; *HMGS*, *3-Hydroxy-3-Methylglutaryl-synthase*; *HMGR*, 3-*hydroxy-3-methylglutaryl-CoA reductase*; *MVK*, *mevalonate kinase*; *PMK*, *Phospho-Mevalonate kinase*; *MPDC*, *Diphospho-MVA decarboxylase*; *IPPI*, *Isopentenyl diphosphate isomerase*; *FPS*, *farnesyl diphosphate synthase*; *SaSSY*, *Santalum album santalene synthase*; *CPR*, *cytochrome P450 reductase*; HMG-CoA, 3-Hydroxy-3-Methylglutaryl-CoA; MVA, Mevalonic acid; MVP, Mevalonic acid-5-phosphate; MVPP, Mevalonic acid-5-diphosphate; IPP, Isopentenyl diphosphate; DMAPP, Dimethylallyl diphosphate; FPP, farnesyl diphosphate.

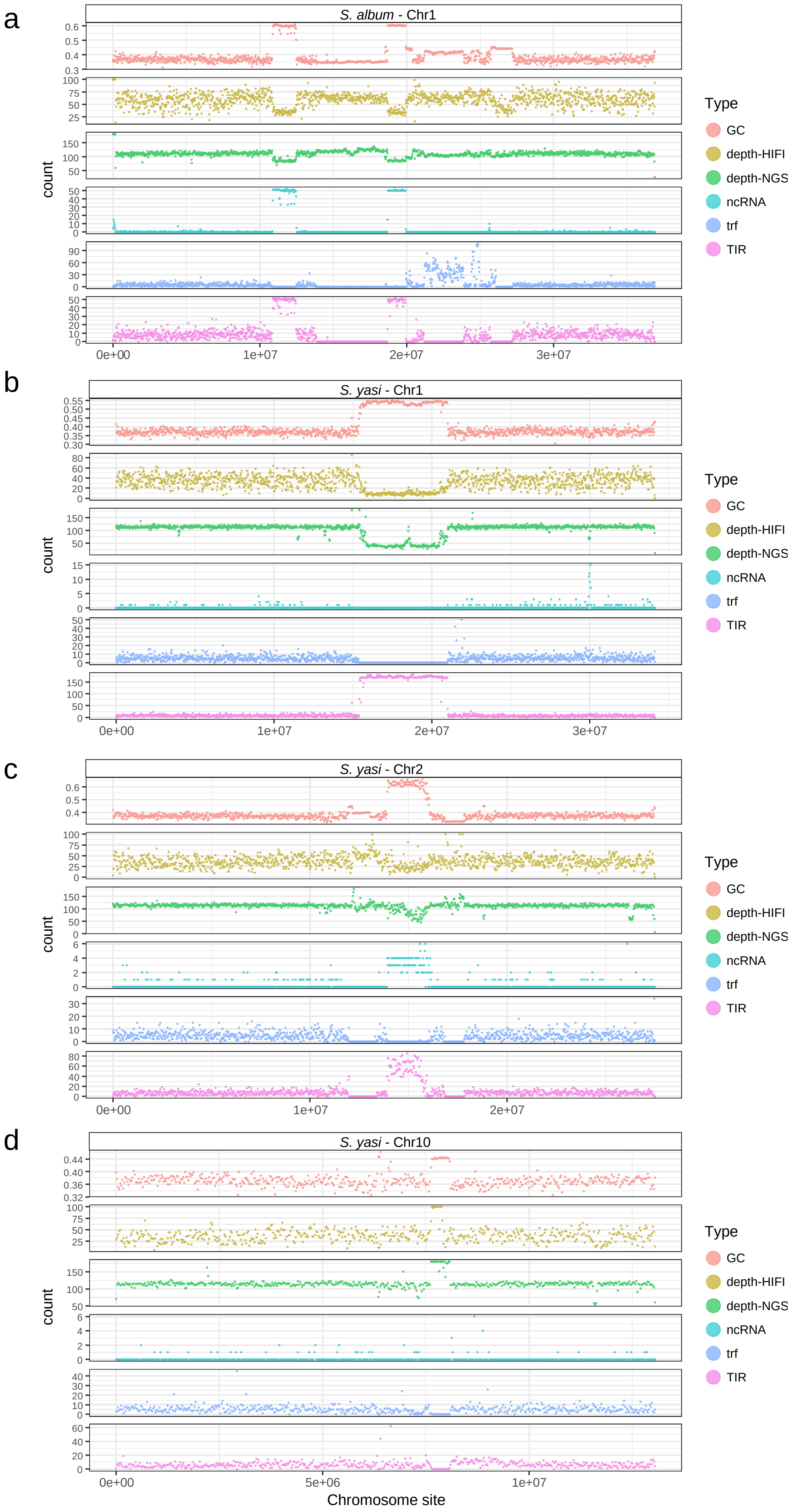

Supplementary Figure 10: Distribution of GC content, depth of Hifi reads, depth of short reads, ncRNA, trf and TIR repeats, in (a) Chr1 of *S. album*, (b) Chr1, (c) Chr2, (d) Chr10 in *S. yasi*, respectively. These metrics were calculated in 25 Kbp windows.

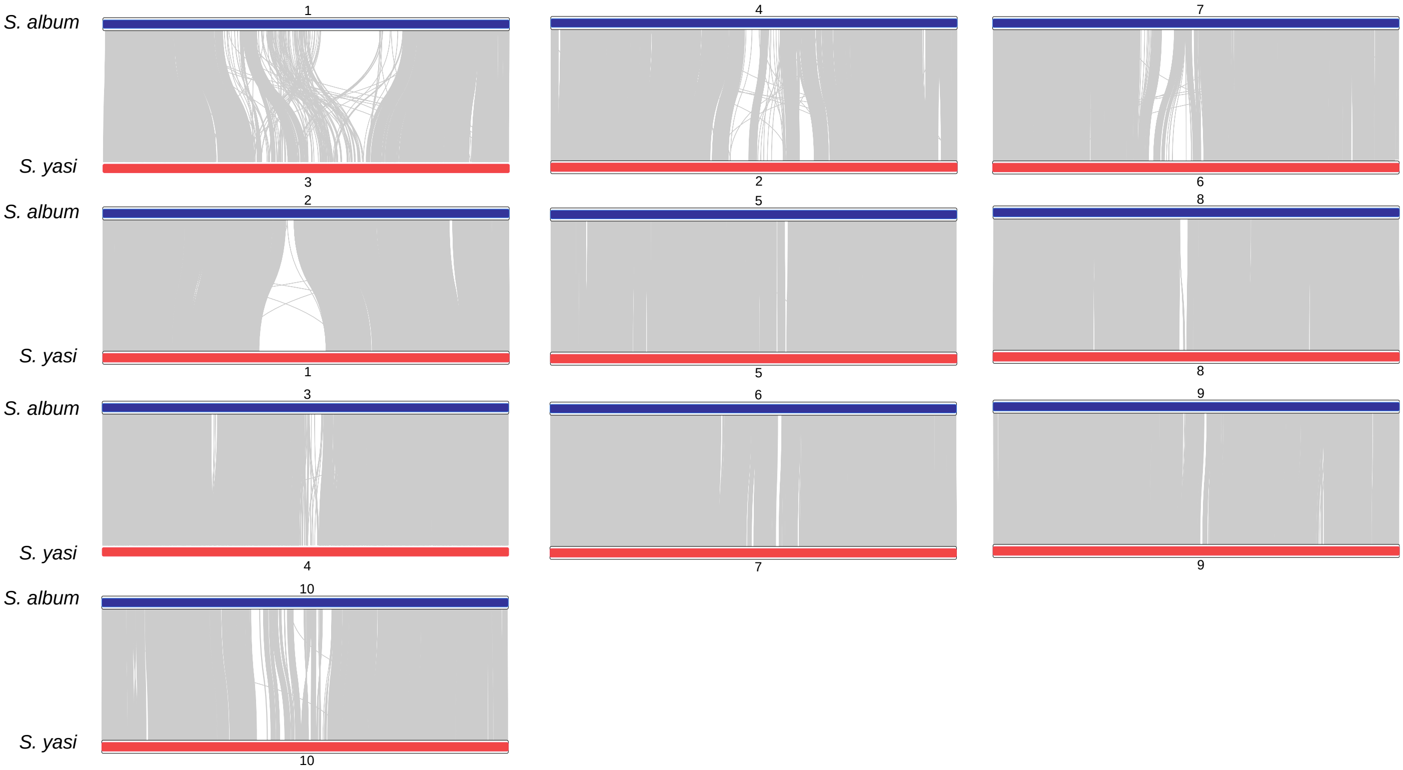

Supplementary Figure 11: Collinearity of genomes by chromosome between *S. album* and *S. yasi*

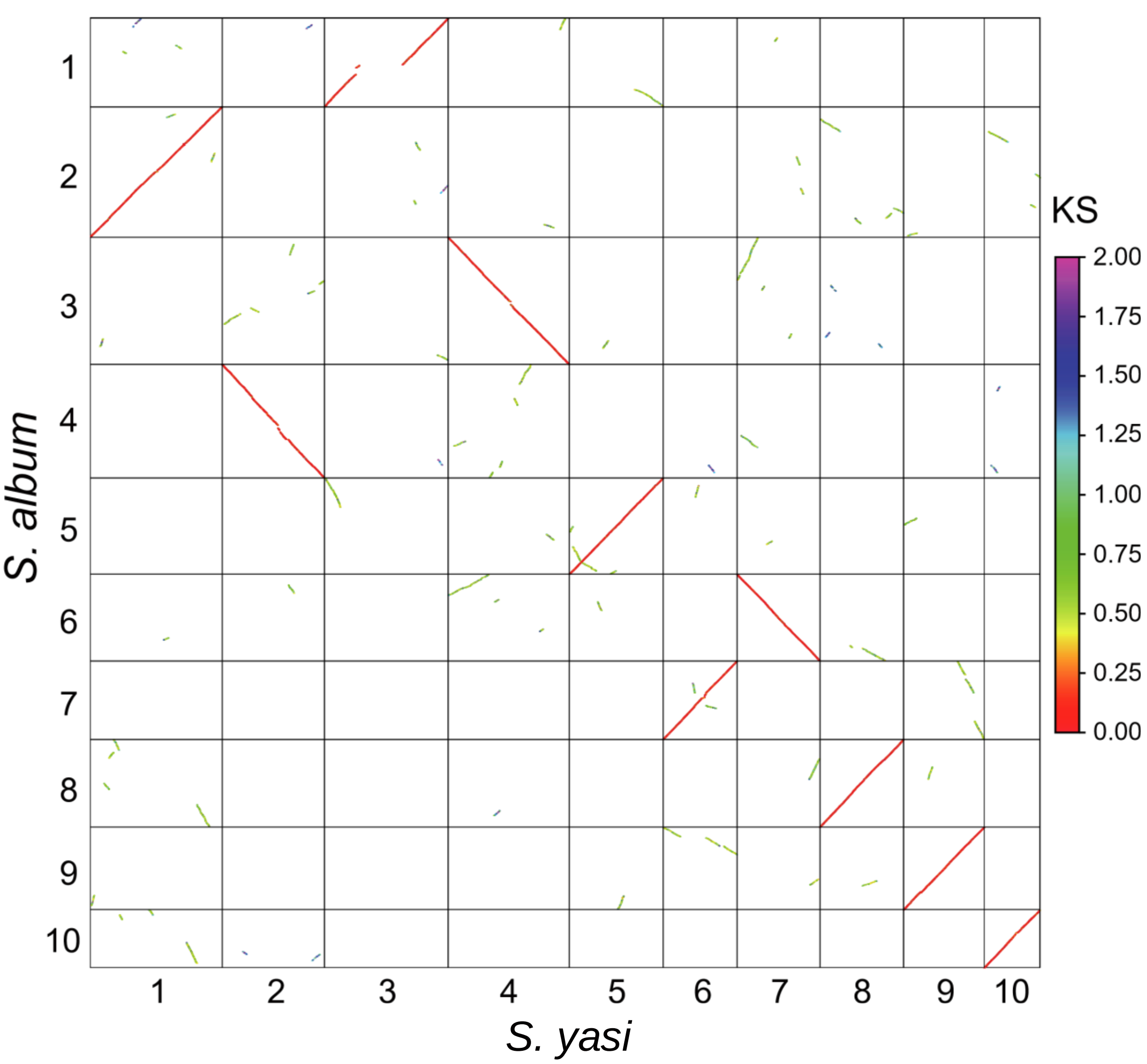

Supplementary Figure 12: Collinearity of genes by chromosome between *S. album* and *S. yasi*. Points representing homologous pair locations were colored according to the Ks color scale

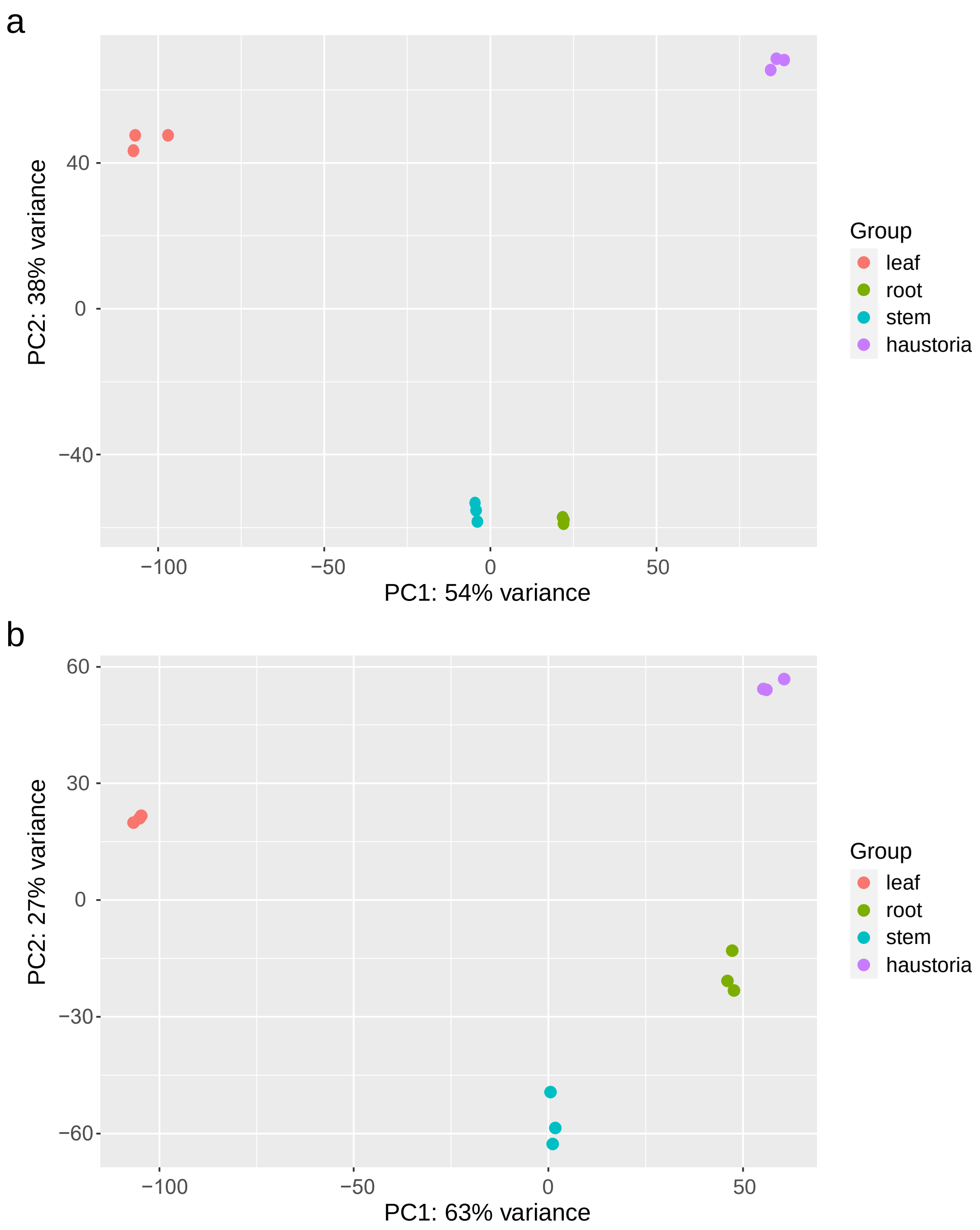

Supplementary Figure 13: PCA of *S. album* (a), and *S. yasi* (b).

Supplemental Table

Supplementary Table 1: DNA data used for genome assembly.

	S. album	*S. yasi*
MGI paired-end library		
ReadNum	93,224,679x2	97,741,089x2
BaseCount(bp)	13,890,822,077;13,865,896,757	14,572,455,835;14,572,455,835
ReadLength(bp)	149;148	149;149
Q20(%)	97.5;97.8	97.7;97.7
Q30(%)	90.4;90.6	90.9;90.9
GC Content(%)	37.9;37.8	38.4;38.4
MGI paired-end library without organelle		
ReadNum	78,134,603x2	84,614,385x2
BaseCount(bp)	11,642,055,847	12,607,543,365
ReadLength(bp)	149;148	149;149
PacBio HiFi long reads		
Total bases(Gb)	14.52	9.97
Total_number	807,901	461,728
Minimum length(bp)	50	79
Average length(bp)	17,975	21,588
Maximum length(bp)	50,382	50,570
N50(bp)	17,844	22,167
Average accuracy	0.99797	0.99748
Average passes	9	8

Supplementary Table 2: Statistics of *S. album* and *S. yasi* genome size, heterozygosity, and repeat ratio

Sample	K-mer number	K-mer Depth	Genome Size(Mb)	Heterozygous Ratio (%)	Repeat(%)
S. album	20,761,079,652	90	217.24	0.42	30.47
S. yasi	22,494,624,417	100	213.52	0.37	30.75

Supplementary Table 3: Statistics of *S. album* and *S. yasi* genome assemblies

Sample	data	software	Total length (Mb)	Chromosome length (Mb)	Total number	Max length(bp)	Contig n50(bp)	Scaffold n50(bp)	BUSCO
S. album	HiFi	HiFiasm	258.27	-	632	18,191,935	9,919,597	-	92.50%
	hic	juicebox	258.29	219.57	608	36,921,381	-	18,339,279	
		purge_dups	229.70	-	54	36,921,381	-	26,521,657	
		pilon	229.59	219.49	54	36,917,765	10,641,032	26,507,775	96.50%
S. yasi	HiFi	HiFiasm	273.90	-	698	10,669,267	2,784,782	-	91.70%
	hic	juicebox	273.94	213.29	609	34,145,193	-	17,496,075	
		purge_dups	232.75	-	66	34,145,193	-	18,169,195	
		pilon	232.64	213.19	66	34,129,967	3,632,663	18,160,053	96.70%

Supplementary Table 4: Repeat sequence prediction in *M. oleifera*

Class	Count	bpMasked	%masked	
LTR	Copia	413,923	363,052,481	24.06%
	Gypsy	360,009	441,491,908	29.26%
	unknown	301,267	187,141,877	12.40%
TIR	CACTA	101,698	37,153,618	2.46%
	Mutator	174,092	55,341,993	3.67%
	PIF_Harbinger	29,726	9,981,272	0.66%
	Tc1_Mariner	16,303	5,477,197	0.36%
	hAT	49,289	17,244,993	1.14%
	polinton	0	0	0.00%
nonLTR	LINE_element	11,298	6,879,903	0.46%
	unknown	766	355,970	0.02%
nonTIR	helitron	41,791	12,352,788	0.82%
repeat_region		189,648	47,871,729	3.17%
Total		1,689,810	1,184,345,729	78.50%
LAI		9		

Supplementary Table 5: Annotations used for EVidenceModeler

		ref gene number	*S. album*	*S. yasi*
	Arabidopsis thaliana	27,444	18,479	18,420
	Beta vulgaris	25,301	18,902	18,796
	Kalanchoe fedtschenkoi	30,964	19,635	19,589
homo	*Malania oleifera*	24,094	24,796	24,685
	Nelumbo nucifera	24,500	19,084	18,943
	Solanum lycopersicum	26,217	19,160	19,126
	Vitis vinifera	26,346	19,214	19,168
	augustus-homo	-	21,581	23,853
de novo	augustus-rna	-	22,428	23,754
	glimmerHMM	-	41,709	45,739
	snap	-	19,088	11,663
transcript	trinity-pasa	-	569,297	524,777
	hisat2-stringtie	-	339,750	349,290
	gene	-	21,673	22,816
total	cds	-	123,868	129,332
	average number of cds per gene	-	5.66	5.26
	mean	-	1,333	1,299
length of gene(cds)	median	-	1,104	1,056
	max	-	16,419	16,395
	busco	-	93.6%	94.3%

Supplementary Table 6: Statistics of *S. album* and *S. yasi* functional annotations

	S. album		*S. yasi*	
	number	ratio	number	ratio
GO	13,329	61.50%	13,027	57.10%
KEGG	7,693	35.50%	7,586	33.25%
Swissprot	15,281	70.51%	15,197	66.61%
Interproscan(Pfam)	20,162(17,065)	93.03%(78.74%)	20,369(16,748)	89.28%(73.40%)
Nr	20,601	95.05%	20,777	91.06%
Total	20,635	95.21%	20,806	91.19%
All	21,673	-	22,816	-

Supplementary Table 7: Statistics of *S. album* and *S. yasi* ncRNA annotations

			S.album		*S.yasi*	
			length	num	length	num
	ribozyme		247	1	247	1
	antisense		2,478	16	2,749	17
	rRNA		3,754,438	7,067	4,213,747	5,973
		5_8S_rRNA	1,609	11	1,362	9
		5S_rRNA	669,709	5,629	517,990	4,354
		LSU_rRNA	2,003,416	671	2,385,622	781
		SSU_rRNA	1,079,704	756	1,308,773	829
Others			12,016	112	14,497	132
	sRNA		409	1	409	1
	tRNA		46,114	614	48,291	641
	miRNA		9,511	71	10,044	76
	riboswitch		115	1	115	1
	snRNA		38,141	329	42,028	346

Supplementary Table 8: The functional annotations of part of uniq genes in *S. album* and *S. yasi*

Pfam number	Pfam annotation	*S. album*	*S. yasi*
PF00078	Reverse transcriptase (RNA-dependent DNA polymerase)	SALChr10.379	SYAChr01.2731, SYAChr02.1395
PF07727	Reverse transcriptase (RNA-dependent DNA polymerase)	-	SYAChr01.150, SYAChr01.389, SYAChr02.1675, SYAChr03.2872, SYAChr03.409
PF13456	Reverse transcriptase-like	-	SYAChr01.946
PF13966	zinc-binding in reverse transcriptase	-	SYAChr01.112, SYAChr02.1400, SYAChr03.563, SYAChr10.431
PF14223	gag-polypeptide of LTR copia-type	SALChr01.1264, SALChr01.1267, SALChr01.1271, SALChr01.1284, SALChr02.1465, SALChr03.1473, SALChr03.1486, SALChr03.1488, SALChr03.1492, SALChr03.1506, SALChr03.1517, SALChr03.1521, SALChr04.1385, SALChr04.1394, SALChr04.1410, SALChr04.1435, SALChr04.1436, SALChr04.1498, SALChr04.2584, SALChr06.1138, SALChr07.785, SALChr07.803, SALChr07.810, SALChr07.812, SALChr08.925, SALChr10.572, SALChr10.573	

Supplementary Table 9: KEGG path enrichment in expanded and contracted gene families of *S. album* and *S. yasi*

Type	ID	Description	qvalue	Count
S. album-expanded	ko00592	alpha-Linolenic acid metabolism	0.000675	4
	ko00520	Amino sugar and nucleotide sugar metabolism	7.90E-05	11
	ko00199	Cytochrome P450	0.000116	7
	ko03032	DNA replication proteins	0.001085	5
	ko99980	Enzymes with EC numbers	6.60E-12	32
	ko00480	Glutathione metabolism	6.60E-12	14
	ko00630	Glyoxylate and dicarboxylate metabolism	0.019859	4
	ko04010	MAPK signaling pathway	9.62E-12	25
	ko03015	mRNA surveillance pathway	0.019859	6
	ko00910	Nitrogen metabolism	2.97E-05	6
	ko00190	Oxidative phosphorylation	0.000221	12
	ko00040	Pentose and glucuronate interconversions	8.54E-06	15
	ko00940	Phenylpropanoid biosynthesis	0.000168	10
	ko04141	Protein processing in endoplasmic reticulum	0.028639	8
	ko03009	Ribosome biogenesis	0.002643	10
	ko05203	Viral carcinogenesis	1.37E-07	8
S. album-contracted	ko00904	Diterpenoid biosynthesis	0.002325	4
	ko04010	MAPK signaling pathway	0.00011	11
	ko00902	Monoterpenoid biosynthesis	2.90E-13	12
	ko04621	NOD-like receptor signaling pathway	0.066221	2
	ko00360	Phenylalanine metabolism	0.001059	2
	ko00940	Phenylpropanoid biosynthesis	0.000905	7
	ko03010	Ribosome	0.047087	8
	ko04726	Serotonergic synapse	4.86E-06	4
	ko04130	SNARE interactions in vesicular transport	0.001988	5
	ko00140	Steroid hormone biosynthesis	2.96E-05	6
	ko03021	Transcription machinery	5.49E-06	9
	ko05202	Transcriptional misregulation in cancer	6.62E-07	6
	ko00130	Ubiquinone and other terpenoid-quinone biosynthesis	0.070415	2
S. yasi-expanded	ko00592	alpha-Linolenic acid metabolism	0.002406	4
	ko00999	Biosynthesis of various secondary metabolites - part 1	3.22E-07	5
	ko04812	Cytoskeleton proteins	2.64E-05	11
	ko00904	Diterpenoid biosynthesis	1.16E-05	9
	ko04010	MAPK signaling pathway	0.000886	15
	ko00902	Monoterpenoid biosynthesis	4.69E-43	34
	ko00040	Pentose and glucuronate interconversions	1.16E-05	17
	ko00940	Phenylpropanoid biosynthesis	1.11E-13	24
	ko04075	Plant hormone signal transduction	0.01026	11
	ko04141	Protein processing in endoplasmic reticulum	7.32E-24	45
	ko04014	Ras signaling pathway	0.017193	3
	ko04726	Serotonergic synapse	2.32E-12	8
	ko04130	SNARE interactions in vesicular transport	3.22E-07	15
	ko00140	Steroid hormone biosynthesis	0.017193	4
	ko03021	Transcription machinery	8.25E-09	17
S. yasi-contracted	ko00592	alpha-Linolenic acid metabolism	0.04218	2
	ko00520	Amino sugar and nucleotide sugar metabolism	3.68E-05	9
	ko00942	Anthocyanin biosynthesis	9.68E-08	5
	ko00073	Cutin, suberine and wax biosynthesis	0.011604	3
	ko03032	DNA replication proteins	0.033914	3
	ko99980	Enzymes with EC numbers	5.45E-10	27
	ko00944	Flavone and flavonol biosynthesis	0.024191	1
	ko00480	Glutathione metabolism	3.68E-05	7
	ko00943	Isoflavonoid biosynthesis	0.020545	2
	ko00591	Linoleic acid metabolism	0.008614	2
	ko04010	MAPK signaling pathway	0.000145	13

	ko00190	Oxidative phosphorylation	0.008612	8
	ko00040	Pentose and glucuronate interconversions	0.000457	11
	ko00940	Phenylpropanoid biosynthesis	0.023825	6
	ko04626	Plant-pathogen interaction	0.036141	5
	ko00909	Sesquiterpenoid and triterpenoid biosynthesis	0.036141	2
	ko04071	Sphingolipid signaling pathway	0.033914	2
	ko00140	Steroid hormone biosynthesis	0.007135	4
	ko03000	Transcription factors	2.14E-05	20
	ko00380	Tryptophan metabolism	0.036057	2

Supplementary Table 10: KEGG path enrichment in contracted gene families of specific gene families in *M. oleifera*

Term Name	Main Class	Gene Hits In Selected Set	All Genes In Selected Set	Gene Hits In Back ground	All Genes In Background	p-value	enrich Factor	corrected p-value (BH method)
04040 Ion channels	A09180 Brite Hierarchies	5	361	51	9741	0.0397	2.6454	0.2908
03020 RNA polymerase	A09120 Genetic Information Processing	6	361	47	9741	0.0074	3.4447	0.0949
00030 Pentose phosphate pathway	A09100 Metabolism	7	361	53	9741	0.0032	3.5638	0.0450
00051 Fructose and mannose metabolism	A09100 Metabolism	8	361	57	9741	0.0011	3.7871	0.0190
00195 Photosynthesis	A09100 Metabolism	10	361	72	9741	0.0003	3.7477	0.0076
00460 Cyanoamino acid metabolism	A09100 Metabolism	10	361	72	9741	0.0003	3.7477	0.0076
00194 Photosynthesis proteins	A09180 Brite Hierarchies	10	361	86	9741	0.0012	3.1376	0.0192
00500 Starch and sucrose metabolism	A09100 Metabolism	13	361	181	9741	0.0171	1.9380	0.1750
03021 Transcription machinery	A09180 Brite Hierarchies	16	361	261	9741	0.0334	1.6542	0.2570
01009 Protein phosphatases and associated proteins	A09180 Brite Hierarchies	19	361	286	9741	0.0100	1.7926	0.1099
00940 Phenylpropanoid biosynthesis	A09100 Metabolism	24	361	184	9741	0.0000	3.5196	0.0000
01002 Peptidases and inhibitors	A09180 Brite Hierarchies	24	361	388	9741	0.0096	1.6691	0.1136
00190 Oxidative phosphorylation	A09100 Metabolism	26	361	145	9741	0.0000	4.8384	0.0000
B 09110 Biosynthesis of other secondary metabolites	A09100 Metabolism	30	361	372	9741	0.0000	2.1761	0.0019
03029 Mitochondrial biogenesis	A09180 Brite Hierarchies	32	361	462	9741	0.0005	1.8690	0.0090
02000 Transporters	A09180 Brite Hierarchies	32	361	601	9741	0.0241	1.4367	0.2318
B 09102 Energy metabolism	A09100 Metabolism	39	361	351	9741	0.0000	2.9982	0.0000
B 09181 Protein families: metabolism	A09180 Brite Hierarchies	85	361	1899	9741	0.0299	1.2078	0.2713
A09100 Metabolism	A09100 Metabolism	130	361	2665	9741	0.0002	1.3163	0.0048

Supplementary Table 11: Statistics of solo/intact ratio in three Santalales species

	solo	intact	ratio
M. oleifera	187,941	46,885	4.01
S. album	4,340	935	4.64
S. yasi	3,945	665	5.93

Supplementary Table 12: KEGG enrichment of part upregulated genes in *S. album*

ID	Swissprot annotation	Pfam number	Pfam annotation	Nr annotation
SALChr01.606	Probable pectinesterase/pectinesterase inhibitor 51;Includes:Pectinesterase inhibitor 51;Includes:Pectinesterase 51;Short=PE 51;EC=3.1.1.11;Short=AtPME51;	PF01095	Pectinesterase	probable pectinesterase/pectinesterase inhibitor 51 isoform X1 [Jatropha curcas]
SALChr01.715	Pectinesterase 2;Short=PE 2;EC=3.1.1.11;	PF01095	Pectinesterase	pectinesterase 2 isoform X2 [Arachis duranensis]; pectinesterase 2 [Arachis hypogaea]; Pectinesterase [Arachis hypogaea]; hypothetical protein Ahy_A02g005443 [Arachis hypogaea]
SALChr02.2019	Pectinesterase 2;Short=PE 2;EC=3.1.1.11;	PF04043	Plant invertase/pectin methylesterase inhibitor	hypothetical protein F0562_014753 [Nyssa sinensis]
SALChr02.264	Probable pectinesterase/pectinesterase inhibitor 51;Includes:Pectinesterase inhibitor 51;Includes:Pectinesterase 51;Short=PE 51;EC=3.1.1.11;Short=AtPME51;	PF04043	Plant invertase/pectin methylesterase inhibitor	probable pectinesterase/pectinesterase inhibitor 51 [Punica granatum]; hypothetical protein CDL15_Pgr017355 [Punica granatum]
SALChr02.2928	Pectinesterase/pectinesterase inhibitor 18;Pectinesterase inhibitor 18;Bifunctional pectinesterase 18/rRNA N-glycosylase;Short=PE 18;EC=3.1.1.11;EC=3.2.2.22;Short=AtPME4;	PF01095	Pectinesterase	pectinesterase [Juglans regia]; hypothetical protein F2P56_006615 [Juglans regia]
SALChr03.1323	Probable pectinesterase/pectinesterase inhibitor 59;Includes:Pectinesterase inhibitor 59;Includes:Pectinesterase 59;Short=PE 59;EC=3.1.1.11;Short=AtPME59;	PF04043	Plant invertase/pectin methylesterase inhibitor	probable pectinesterase/pectinesterase inhibitor 33 [Vitis riparia]
SALChr03.2480	Probable pectinesterase/pectinesterase inhibitor 40;Includes:Pectinesterase inhibitor 40;Includes:Pectinesterase 40;Short=PE 40;EC=3.1.1.11;Short=AtPME40;	PF01095	Pectinesterase	hypothetical protein FH972_005885 [Carpinus fangiana]
SALChr04.89	Probable pectinesterase/pectinesterase inhibitor 21;Includes:Pectinesterase inhibitor 21;Includes:Pectinesterase 21;Short=PE 21;EC=3.1.1.11;Short=AtPME21;	PF01095	Pectinesterase	hypothetical protein HHK36_032636 [Tetracentron sinense]
SALChr05.1890	Putative pectinesterase/pectinesterase inhibitor 24;Includes:Pectinesterase inhibitor 24;Includes:Pectinesterase 24;Short=PE 24;EC=3.1.1.11;Short=AtPME24;	PF04043	Plant invertase/pectin methylesterase inhibitor	hypothetical protein HHK36_021948 [Tetracentron sinense]
SALChr05.1891	Probable pectinesterase/pectinesterase inhibitor 25;Includes:Pectinesterase inhibitor 25;Includes:Pectinesterase 25;Short=PE 25;EC=3.1.1.11;Short=AtPME25;	PF01095	Pectinesterase	Pectinesterase [Macleaya cordata]
SALChr05.1892	Probable pectinesterase/pectinesterase inhibitor 47;Includes:Pectinesterase inhibitor 47;Includes:Pectinesterase 47;Short=PE 47;EC=3.1.1.11;Short=AtPME47;	PF04043	Plant invertase/pectin methylesterase inhibitor	unnamed protein product [Coffea canephora]
SALChr05.375	Probable pectinesterase 53;Short=PE 53;EC=3.1.1.11;Short=AtPME53;	PF01095	Pectinesterase	hypothetical protein EZV62_012842 [Acer yangbiense]
SALChr06.1592	Pectinesterase 2;Short=PE 2;EC=3.1.1.11;	PF01095	Pectinesterase	pectinesterase 2-like [Coffea arabica]; pectinesterase 2-like [Coffea eugenioides]
SALChr06.576	Probable pectinesterase/pectinesterase inhibitor 12;Includes:Pectinesterase inhibitor 12;Includes:Pectinesterase 12;Short=PE 12;EC=3.1.1.11;Short=AtPME12;	PF04043	Plant invertase/pectin methylesterase inhibitor	probable pectinesterase/pectinesterase inhibitor 12 [Herrania umbratica]
SALChr10.228	Pectinesterase 3;Short=PE 3;EC=3.1.1.11;	PF04043	Plant invertase/pectin methylesterase inhibitor	PREDICTED: pectinesterase 3-like [Populus euphratica]
SALChr10.229	Pectinesterase 3;Short=PE 3;EC=3.1.1.11;	PF04043	Plant invertase/pectin methylesterase inhibitor	hypothetical protein FH972_003460 [Carpinus fangiana]
SALChr02.2019	Pectinesterase 2;Short=PE 2;EC=3.1.1.11;	PF04043	Plant invertase/pectin methylesterase inhibitor	hypothetical protein F0562_014753 [Nyssa sinensis]
SALChr02.264	Probable pectinesterase/pectinesterase inhibitor 51;Includes:Pectinesterase inhibitor 51;Includes:Pectinesterase 51;Short=PE 51;EC=3.1.1.11;Short=AtPME51;	PF04043	Plant invertase/pectin methylesterase inhibitor	probable pectinesterase/pectinesterase inhibitor 51 [Punica granatum]; hypothetical protein CDL15_Pgr017355 [Punica granatum]
SALChr03.1323	Probable pectinesterase/pectinesterase inhibitor 59;Includes:Pectinesterase inhibitor 59;Includes:Pectinesterase 59;Short=PE 59;EC=3.1.1.11;Short=AtPME59;	PF04043	Plant invertase/pectin methylesterase inhibitor	probable pectinesterase/pectinesterase inhibitor 33 [Vitis riparia]
SALChr05.1890	Putative pectinesterase/pectinesterase inhibitor 24;Includes:Pectinesterase inhibitor 24;Includes:Pectinesterase 24;Short=PE 24;EC=3.1.1.11;Short=AtPME24;	PF04043	Plant invertase/pectin methylesterase inhibitor	hypothetical protein HHK36_021948 [Tetracentron sinense]
SALChr05.1892	Probable pectinesterase/pectinesterase inhibitor 47;Includes:Pectinesterase inhibitor 47;Includes:Pectinesterase 47;Short=PE 47;EC=3.1.1.11;Short=AtPME47;	PF04043	Plant invertase/pectin methylesterase inhibitor	unnamed protein product [Coffea canephora]
SALChr06.576	Probable pectinesterase/pectinesterase inhibitor 12;Includes:Pectinesterase inhibitor 12;Includes:Pectinesterase 12;Short=PE 12;EC=3.1.1.11;Short=AtPME12;	PF04043	Plant invertase/pectin methylesterase inhibitor	probable pectinesterase/pectinesterase inhibitor 12 [Herrania umbratica]

SALChr10.228	Pectinesterase 3;Short=PE 3;EC=3.1.1.11;	PF04043	Plant invertase/pectin methylesterase inhibitor	PREDICTED: pectinesterase 3-like [Populus euphratica]
SALChr10.229	Pectinesterase 3;Short=PE 3;EC=3.1.1.11;	PF04043	Plant invertase/pectin methylesterase inhibitor	hypothetical protein FH972_003460 [Carpinus fangiana]
SALChr01.319	Xyloglucan endotransglucosylase/hydrolase protein 22;Short=At-XTH22;Short=XTH-22;EC=2.4.1.207;	PF00722	Glycosyl hydrolases family 16	Xyloglucan endotransglucosylase/hydrolase [Trema orientale]
SALChr05.1069	Probable xyloglucan endotransglucosylase/hydrolase protein 23;Short=At-XTH23;Short=XTH-23;EC=2.4.1.207;	PF00722	Glycosyl hydrolases family 16	probable xyloglucan endotransglucosylase/hydrolase protein 23 [Vitis riparia]
SALChr05.1070	Probable xyloglucan endotransglucosylase/hydrolase protein 23;Short=At-XTH23;Short=XTH-23;EC=2.4.1.207;	PF00722	Glycosyl hydrolases family 16	putative xyloglucan endotransglucosylase/hydrolase protein 23 [Mucuna pruriens]
SALChr05.656	Xyloglucan endotransglucosylase/hydrolase protein 22;Short=At-XTH22;Short=XTH-22;EC=2.4.1.207;	PF06955	Xyloglucan endotransglycosylase (XET) C-terminus	hypothetical protein HYC85_003792 [Camellia sinensis]

Supplementary Table 13: KEGG enrichment of part upregulated genes in *S. yasi*

ID	Swissprot annotation	Pfam number	Pfam annotation	Nr annotation
SYAChr02.1793	Cytokinin dehydrogenase 5;EC=1.5.99.12;Short=AtCKX5;Short=AtCKX6;Short=CKO5;	PF09265	Cytokinin dehydrogenase 1, FAD and cytokinin binding	Cytokinin dehydrogenase [Actinidia chinensis var. chinensis]
SYAChr05.1925	Cytokinin dehydrogenase 3;EC=1.5.99.12;Short=AtCKX3;Short=CKO 3;	PF01565	FAD binding domain	PREDICTED: cytokinin dehydrogenase 3 [Vitis vinifera]; cytokinin dehydrogenase 3c [Vitis vinifera]
SYAChr06.1679	Cytokinin dehydrogenase 7;EC=1.5.99.12;Short=AtCKX5;Short=AtCKX7;Short=CKO7;	PF09265	Cytokinin dehydrogenase 1, FAD and cytokinin binding	hypothetical protein CMV_005861 [Castanea mollissima]
SYAChr07.1696	Cytokinin dehydrogenase 6;EC=1.5.99.12;Short=AtCKX6;Short=AtCKX7;Short=CKO6;	PF09265	Cytokinin dehydrogenase 1, FAD and cytokinin binding	Cytokinin dehydrogenase [Actinidia chinensis var. chinensis]
SYAChr07.639	Cytokinin dehydrogenase 5;EC=1.5.99.12;Short=AtCKX5;Short=AtCKX6;Short=CKO5;	PF09265	Cytokinin dehydrogenase 1, FAD and cytokinin binding	cytokinin dehydrogenase 5 [Ricinus communis]; gulonolactone oxidase, putative [Ricinus communis]
SYAChr02.1783	Auxin response factor 5;	PF02362	B3 DNA binding domain	auxin response factor 5 [Vitis riparia]
SYAChr04.1105	Auxin response factor 5;	PF06507	Auxin response factor	auxin response factor 5-like [Lactuca sativa]
SYAChr09.61	1-aminocyclopropane-1-carboxylate oxidase;Short=ACC oxidase;EC=1.14.17.4;Short=EFE;	PF14226	non-haem dioxygenase in morphine synthesis N-terminal	ethylene-forming enzyme [Actinidia rufa]
SYAChr09.748	1-aminocyclopropane-1-carboxylate oxidase 3;Short=ACC oxidase 3;EC=1.14.17.4;Short=EFE;	PF03171	2OG-Fe(II) oxygenase superfamily	RecName: Full=1-aminocyclopropane-1-carboxylate oxidase 3; Short=ACC oxidase 3; AltName: Full=Ethylene-forming enzyme; Short=EFE [Petunia x hybrida]; 1-aminocyclopropane-1-carboxylate oxidase [Petunia x hybrida]

Supplementary Table 14: Repeat sequence prediction in *S. album* and *S. yasi* (RepeatMasker and RepeatModeler software)

	S.album			*S.yasi*		
Assembly size （Mb）	229.59			232.62		
	Count	bpMasked	%masked	Count	bpMasked	%masked
Retroelements	20,718	17,372,258	7.57	17,282	12,254,142	5.27
SINEs:	-	-	-	-	-	-
Penelope	-	-	-	-	-	-
LINEs:	7,747	2,908,981	1.27	7,536	2,700,518	1.16
CRE/SLACS	-	-	-	-	-	-
L2/CR1/Rex	-	-	-	-	-	-
R1/LOA/Jockey	-	-	-	-	-	-
R2/R4/NeSL	-	-	-	-	-	-
RTE/Bov-B	1,631	333,422	0.15	2,331	535,809	0.23
L1/CIN4	6,116	2,575,559	1.12	5,205	2,164,709	0.93
LTR elements:	12,971	14,463,277	6.30	9,746	9,553,624	4.11
BEL/Pao	-	-	-	39	20,663	0.01
Ty1/Copia	9,059	11,859,264	5.17	7,057	7,235,162	3.11
Gypsy/DIRS1	3,149	1,966,796	0.86	2,029	1,707,379	0.73
Retroviral	-	-	-	-	-	-
DNA transposons	3,109	1,816,685	0.79	2,949	1,585,339	0.68
hobo-Activator	1,080	431,554	0.19	655	302,374	0.13
Tc1-IS630-Pogo	-	-	-	-	-	-
En-Spm	-	-	-	-	-	-
MuDR-IS905	-	-	-	-	-	-
PiggyBac	-	-	-	-	-	-
Tourist/Harbinger	158	76,355	0.03	181	83,095	0.04
Other (Mirage, P-element, Transib)	-	-	-	-	-	-
Rolling-circles	955	258,760	0.11	171	153,341	0.07
Unclassified:	216,939	58,452,925	25.46	240,941	69,138,011	29.72
Total interspersed	repeats:	77,641,868	33.82	repeats:	82,977,492	35.67

Supplementary Table 15: Data used for phylogenetic reconstruction

Species	Data source
Nelumbo nucifera	Chinese Lotus 1.1 (NCBI)
Trochodendron aralioides	PRJEB32669 (gigadb)
Kalanchoe fedtschenkoi	Kalanchoe fedtschenkoi v1.1 (Phytozome)
Lupinus albus	La_Amiga3.1 (NCBI)
Aquilaria sinensis	PRJNA556948 (gigadb)
Arabidopsis thaliana	TAIR10.1 (NCBI)
Malania oleifera	PRJNA472200 (gigadb)
Nyssa sinensis	ASM863837v1 (NCBI)
Rhododendron simsii	ASM1428224v1 (NCBI)
Solanum lycopersicum	SL3.0 (NCBI)
Daucus carota	ASM162521v1 (NCBI)
Helianthus annuus	HanXRQr1.0 (NCBI)
Vitis vinifera	Vvinifera_457_v2.1 (Phytozome)
Amborella trichopoda	AT_V6.1 (CoGe)
