# Supplementary material for: Chromosome-level genome assemblies from two sandalwood species provide insights into the evolution of the Santalales
Source: Commun Biol. 2023 Jun 1;6:587. doi: 10.1038/s42003-023-04980-2 (PMC10235099; doi:10.1038/s42003-023-04980-2)
Supplement: Supplementary file 2 — Description of Additional Supplementary Files [file 42003_2023_4980_MOESM2_ESM.pdf]

Description of Additional Supplementary Files

File name: Supplementary Data 1

Description: Statistics of all gene families of 16 species.

File name: Supplementary Data 2

Description: Ks calculation results of paralogs in *S. album*.

File name: Supplementary Data 3

Description: Ks calculation results of paralogs in *S. yasi*.

File name: Supplementary Data 4

Description: Ks calculation results of paralogs in *M. oleifera*.

File name: Supplementary Data 5

Description: Ks calculation results of paralogs in *V. vinifera*.

File name: Supplementary Data 6

Description: Supplementary Data 6: Insert time of LTR in *S. album*.

File name: Supplementary Data 7

Description: Supplementary Data 6: Insert time of LTR in *S. yasi*.

File name: Supplementary Data 8

Description: Supplementary Data 6: Insert time of LTR in *M. oleifera*.
